# Biocompatible Glues: Recent Progress and Emerging Frontiers in Surgical Adhesion

**DOI:** 10.3390/polym17131749

**Published:** 2025-06-24

**Authors:** Marine Boursier, Yves Bayon, Claire Negrell, Julien Pinaud, Sylvain Caillol

**Affiliations:** 1 ICGM, Univ Montpellier, CNRS, ENSCM, 34090 Montpellier, France; marine.boursier2702@gmail.com (M.B.); claire.negrell@enscm.fr (C.N.); julien.pinaud@umontpellier.fr (J.P.); 2 Medtronic—Sofradim Production, F-01600 Trévoux, France; yves.bayon@medtronic.com

**Keywords:** biobased polymers, biodegradable polymers, polyurethanes, adhesion, surgical glues

## Abstract

Surgical adhesives and glues have gained significant attention in the medical field due to their potential to replace traditional sutures and staples in various surgical applications. This review explores the evolution of biocompatible adhesives, focusing on their chemical composition, mechanical properties, and biocompatibility. We discuss the key challenges in developing these materials, including their adhesive strength, degradation rate, and tissue compatibility. The article also delves into regulatory frameworks governing their use in clinical settings and highlights the ongoing innovations aimed at enhancing their performance and safety. Finally, the review examines the current trends in the development of next-generation surgical adhesives, with an emphasis on environmentally friendly and bioresorbable options. The importance of multidisciplinary collaboration in advancing these materials for clinical use is also underscored.

## 1. Introduction

Since ancient times, various methods have been employed in the treatment of wounds and surgical interventions to close tissues or affix both natural and synthetic implantable materials, such as surgical meshes. Staples, absorbable sutures, and more recently, steri-strips^TM^ are among the most commonly used tools for wound closure. Similarly, screws or sutures are traditionally used to anchor prosthetic materials to internal tissues. These techniques remain widely practiced due to their ease of use and high tensile strength. However, from a clinical perspective, they are often poorly adapted to modern surgical demands—particularly in minimally invasive and laparoscopic procedures—due to several technological shortcomings. They are invasive, time-consuming, often require removal through a second intervention, and may lead to leakage of biological fluids [1]. Moreover, they can cause pain, scarring, delayed healing due to poor tissue integration, and, in severe cases, infections. Such drawbacks directly conflict with key clinical goals of reducing surgical trauma, improving postoperative recovery, and enabling seamless integration of biomaterials [2,3,4].

**Scheme 1:**
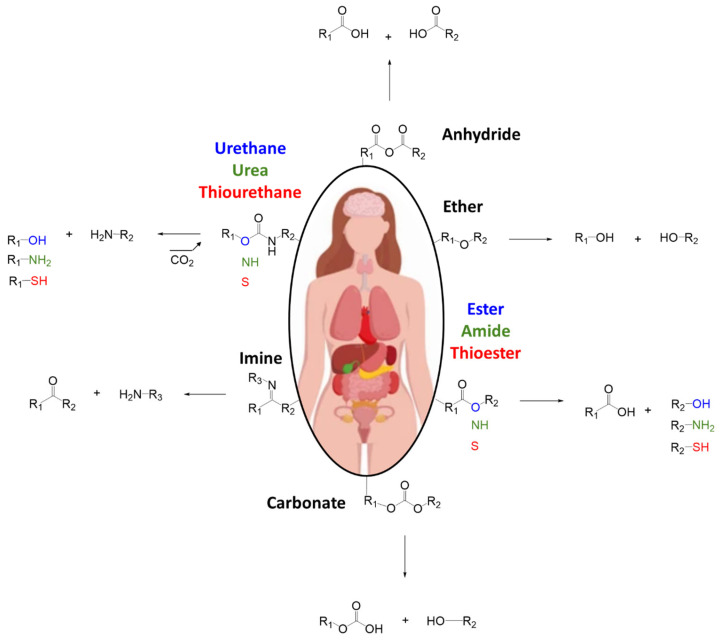
Physio-degradation of chemical bonds with products on degradation.

**Figure 1:**
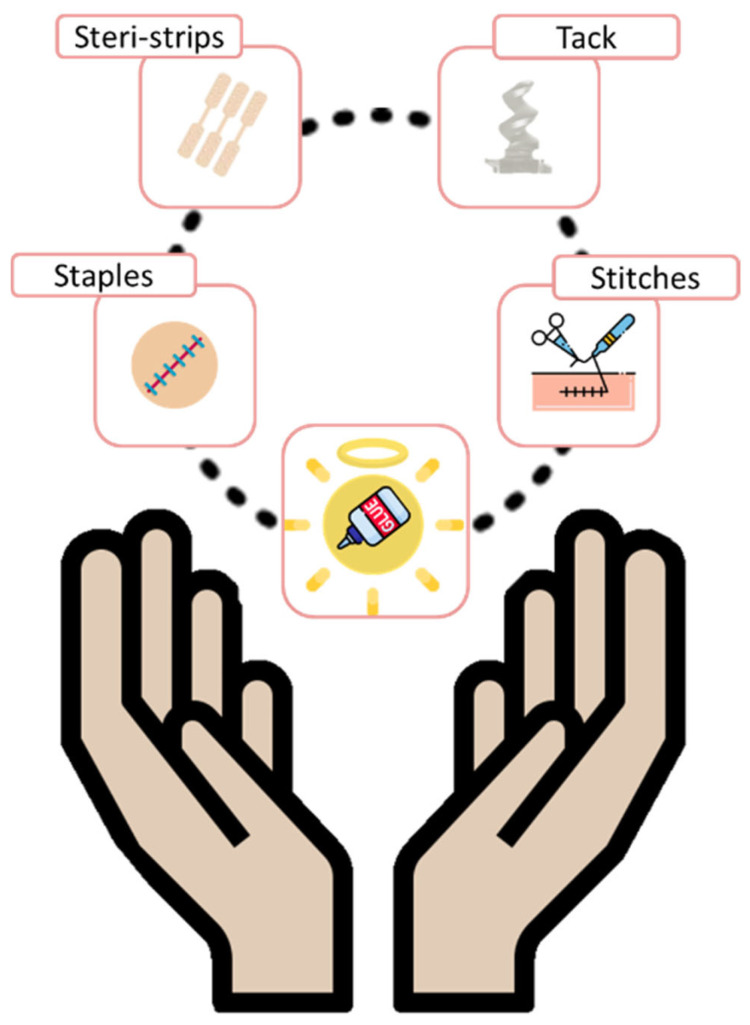
Overview of the different techniques for wound management and surgery.

In this context, surgical adhesives have emerged as promising alternatives capable of overcoming these challenges (Figure 1). These glues are non-invasive, easy to use, and fast to apply [5]. Additionally, some formulations serve dual purposes as hemostatic agents to help control bleeding [6]. Adhesives are defined as non-metallic materials capable of creating durable bonds between surfaces. Surgical adhesives, in particular, are designed to bond tissues or affix implantable biomaterials, and can be composed of a wide range of compounds, including synthetic polymers and natural materials of animal or plant origin [7,8]. The chemical functionalities susceptible to physiological degradation are shown in Scheme 1. The physiological degradation of surgical adhesives is governed by a combination of hydrolytic, oxidative, and enzymatic mechanisms, which act in parallel or sequentially depending on the chemical structure of the adhesive and the specific biological environment. Hydrolytic degradation primarily affects ester, amide, and urethane bonds through nucleophilic attack by water molecules, a process that is significantly influenced by local pH. While ester bonds are typically labile in neutral to slightly basic environments (e.g., blood plasma), amide hydrolysis is more prominent in acidic compartments such as lysosomes. In parallel, oxidative degradation targets sulfur-containing groups such as thiols and thioesters, which undergo oxidation in the presence of reactive oxygen species (ROS) such as hydrogen peroxide and superoxide radicals—molecules commonly produced during inflammatory responses or in metabolically active organs such as the liver and lungs. Lastly, enzymatic degradation involves cleavage by specific enzymes, including esterases, proteases, and ureases, that selectively hydrolyze functional groups such as urea, urethane, and peptide-like bonds. These enzymatic processes are organ- and tissue-specific, with higher activity in protease-rich environments such as chronic wounds, the gastrointestinal tract, and the liver. Together, these degradation pathways determine the bioresorption rate and biocompatibility profile of medical adhesives, making their careful design essential for safe and effective clinical applications.

**Figure 2:**
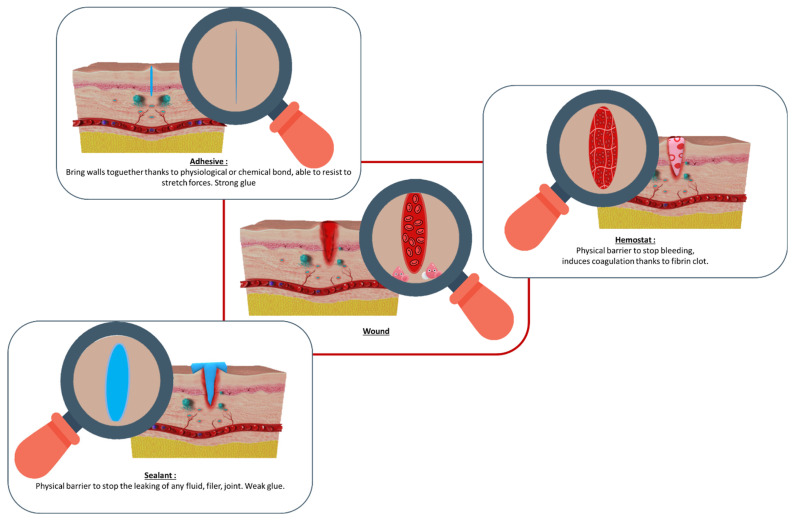
Representation of wound treatment with adhesive, sealant, and hemostat.

Furthermore, certain surgical adhesives exhibit antibacterial properties. These adhesives are typically classified into three major categories (Figure 2) [9]:

- Hemostats: require the presence of blood to initiate clot formation, accelerating hemostasis via mechanical means. They are especially useful in cases of non-suturable hemorrhages.
- Sealants: designed to prevent fluid leakage, they are effective against a variety of bodily fluids beyond blood and are available in both active and flowable formulations.
- Bioadhesives: biocompatible glues used to bond biological materials—either native tissues or synthetic/biological implants—to biological surfaces such as skin, blood vessels, or organs [10].

**Figure 3:**
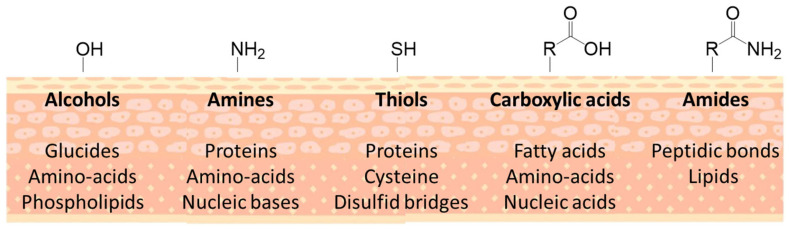
Functional groups and locations in tissues.

Some products, such as fibrin-based adhesives, can exhibit properties characteristic of all three categories. Figure 3 illustrates the main functional groups found on tissue surfaces that participate in adhesive interactions [11,12]. As interest in biomaterials continues to grow, bioadhesion has become a significant area of research [13,14,15,16,17,18,19,20].

**Figure 4:**
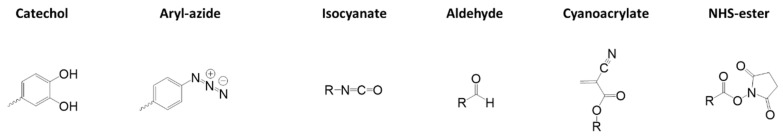
Reactive functions widely used in surgical adhesives.

Bioadhesion can occur through three primary mechanisms: physiological, physical, and chemical interactions. Since blood and bodily fluids can compete with adhesive functional groups and hinder adhesion, it is important that surgical glues employ multiple types of interactions simultaneously. The main reactive groups commonly used in tissue adhesives are shown in Figure 4 [6,11,12,21]. Adhesion typically proceeds in three stages: wetting, polymer swelling, and chain interdiffusion with biological membranes, followed by the formation of chemical bonds between entangled chains [22,23]. From a clinical standpoint, successful adhesives must deliver strong, consistent bonding under real surgical conditions, including the presence of blood, tissue movement, and moisture. To be considered ideal, a surgical adhesive must fulfill the following criteria:

- Biocompatibility: both the adhesive and its degradation products must be safe, non-toxic, and non-immunogenic.
- Ease of application and repositionability: the material should be user-friendly and allow repositioning shortly after application.
- Fast gelation: the adhesive must gel or cure rapidly, even in moist environments containing biological fluids.
- Strong bonding under wet conditions: after crosslinking, the material should exhibit good flexibility and maintain robust adhesion in physiological environments.
- Physiological degradation: ideally, the adhesive should undergo enzymatic or hydrolytic degradation into non-toxic byproducts, which are then safely excreted via the kidneys or liver [24].

Despite their clear advantages, surgical adhesives are not without limitations [25]. Some, such as fibrin-based adhesives, lack sufficient mechanical strength. Others, such as cyanoacrylates, suffer from intrinsic toxicity. Repositioning the adhesive after application is often difficult, and some glues can be destabilized by the presence of blood [3]. Thus, the perfect surgical glue remains elusive, with existing options each presenting a compromise between pros and cons. This underscores a critical technological bottleneck in aligning adhesive performance with the complex biomechanical and biochemical requirements of real-world clinical practice. An ideal surgical adhesive must meet five essential requirements [26,27]:

1. Safety: no toxicity, no disease transmission, and biocompatibility of degradation products.
2. Efficacy: high enough bonding strength to perform reliably in the intended surgical environment.
3. Usability: the material should be easy and quick to apply.
4. Affordability: cost must remain reasonable (ideally under $100 per application) to be competitive in surgical settings.
5. Regulatory Approval: products must obtain formal approval (e.g., from the FDA) for clinical use [28].

Beyond technical development, a major challenge is convincing medical professionals to transition from traditional techniques to these innovative adhesives.

**Figure 5:**
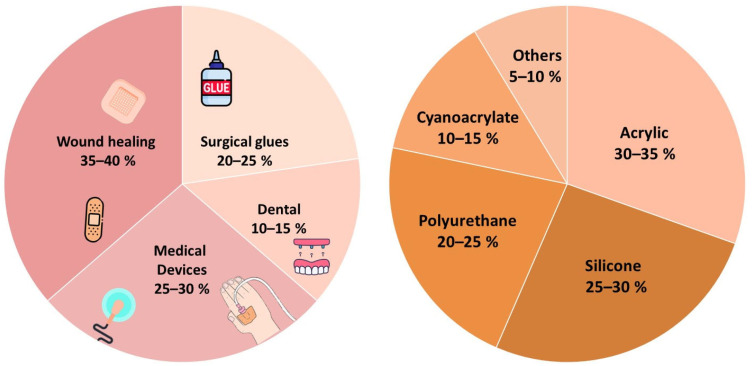
Medical adhesives market distribution by application and by polymer: $9.3B in 2022 [31].

In 2022, the global market for medical adhesives was estimated at approximately $9.3 billion and is projected to grow at a compound annual growth rate of 6–7% through 2027 (Figure 5) [29,30]. Medical adhesives are broadly categorized into four key sectors: implantable adhesives, dental adhesives, adhesives for medical device fixation, and topical skin adhesives.

A 2019 review by Jain and Waikar provided an overview of commercial surgical glues with a focus on their clinical applications but offered limited discussion on structure–property relationships [23]. This review aims to fill that gap by integrating the clinical context with chemical perspectives and by identifying how molecular design can address real surgical limitations. It particularly highlights recent advances in synthetic implantable glues and explores emerging bioadhesion mechanisms. The review is divided into two main parts: the first covers skin pressure-sensitive adhesives (PSAs), while the second delves into wound closure adhesives and surgical glues, with a detailed discussion of both natural-based and synthetic systems.

## 2. Mechanisms of Adhesion

**Figure 6:**
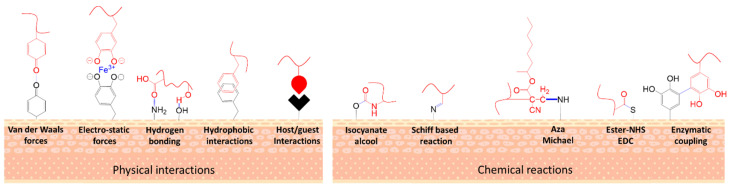
Representation of some physical interactions and chemical reactions.

Understanding the mechanisms by which surgical glues adhere to biological tissues is essential for improving their performance and tailoring them to specific applications. Tissue adhesion is a complex process involving a variety of physicochemical interactions between the adhesive material and the tissue substrate. These interactions can be broadly divided into three main categories: physiological mechanisms, physical interactions (including both interfacial and mechanical phenomena), and chemical bonding (Figure 6) [12,22,32,33,34,35,36,37,38].

### 2.1. Physiological Mechanisms

Physiological adhesion refers to interactions that occur naturally within the body and are often exploited by biologically derived adhesives. These mechanisms are typically based on biological recognition processes such as antigen–antibody interactions, enzyme–substrate specificity, and cellular signaling cascades [39]. A well-known example is fibrin glue, which mimics the body’s natural blood-clotting mechanism. Fibrin glue operates through the thrombin-mediated polymerization of fibrinogen, forming an insoluble fibrin network that stabilizes the wound and promotes hemostasis. This cascade involves a sequence of biological steps, including vasoconstriction, platelet aggregation, fibrin formation, and fibrinolysis during tissue regeneration. Such physiologically inspired adhesives have the advantage of excellent biocompatibility, though they may lack mechanical robustness [40].

### 2.2. Physical Bonds

#### 2.2.1. Physical Interactions

Physical interactions do not involve the formation of covalent bonds but rely on weaker, non-covalent forces that play a critical role in adhesion, especially under wet or complex conditions similar to those encountered in biological systems. The main types of physical interactions include [41,42,43,44]:

- Van der Waals Forces: these are fundamental, short-range interactions that arise from induced or permanent dipoles between molecules. Although individually weak, they become collectively significant when two surfaces are brought into nanometer-scale proximity, enabling effective adhesion. These forces are critical in the adhesion mechanisms of organisms such as geckos, which utilize microscopic fibrillar structures on their feet to maximize contact area and thus enhance van der Waals interactions across the substrate interface.
- Hydrogen Bonds: strong directional interactions formed between hydrogen atoms covalently bound to electronegative atoms (typically oxygen or nitrogen) and lone pairs on other electronegative atoms. These bonds are critical in protein folding and many natural adhesives.
- Electrostatic Interactions: result from attractions between oppositely charged ions or dipoles. The interaction strength depends on ionic concentration, pH, and the dielectric properties of the surrounding medium.
- Hydrophobic Interactions: nonpolar surfaces aggregate in aqueous environments to minimize their exposure to water, leading to adhesion through entropy-driven processes. These interactions are particularly relevant in lipid-rich tissues.

In addition to these interfacial phenomena, two mechanical effects also significantly contribute to physical adhesion:

- Mechanical Interlocking: adhesive penetration into micro- or nano-scale roughness or pores on the tissue surface provides anchoring through physical constraint.
- Polymer Chain Entanglement: long polymer chains from the adhesive can diffuse into the tissue matrix, leading to entanglement and increased interfacial cohesion. This interpenetration enhances load transfer and improves the durability of the adhesive bond.

Diffusion-based mechanisms can also lead to the formation of interpenetrating polymer networks (IPNs), where adhesive molecules interweave with tissue components, creating a cohesive and mechanically strong interface.

#### 2.2.2. Mechanical Interlocking in Surgical Adhesives

**Figure 7:**
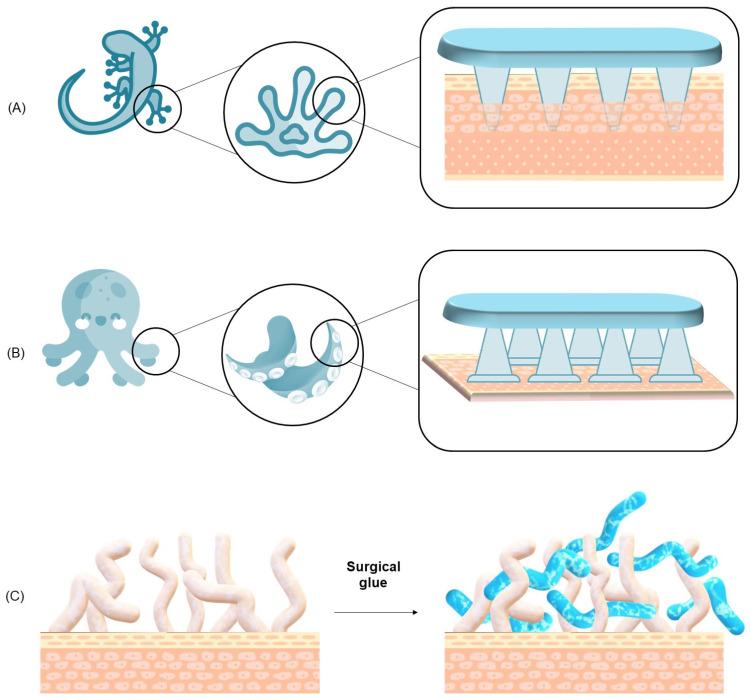
(**A**) Gecko-inspired dry adhesion: The gecko’s footpad exhibits hierarchical fibrillar structures that enable van der Waals interactions. The synthetic analog mimics this with micro/nano-scale projections (~10 µm scale bar) for reversible dry adhesion; (**B**) octopus-inspired suction adhesion: octopus suckers provide strong wet adhesion via controllable suction forces. Biomimetic designs replicate this with micro-suction cups (~100 µm scale bar) for underwater or moist environments; (**C**) worm/mucus-inspired chemical adhesion: mucus-secreting invertebrates adhere via entanglement and interpenetrating networks. Medical adhesives mimic through crosslinked polymer networks (~1 mm scale bar), forming a robust interface upon application of surgical glue.

Adhesion in surgical adhesives arises from several distinct mechanisms, among which mechanical interlocking is particularly important for ensuring effective adhesion to complex and irregular biological substrates. It is essential to distinguish this mechanism from physical adhesion, a term that more accurately refers to non-covalent molecular interactions such as van der Waals forces, hydrogen bonding, and electrostatic attractions—all of which operate at the nanometer scale between closely apposed surfaces. In contrast, mechanical interlocking (also referred to as mechanical anchoring) describes the physical penetration and entanglement of adhesive polymers into the microporous structures or surface irregularities of tissues. This process creates a form of topographical adhesion whereby the adhesive conforms to and becomes entrapped within the contours of the tissue. Biological substrates such as the dermis, fascia, and trabecular bone naturally contain microarchitectures that facilitate this type of anchoring. Unlike chemical bonding mechanisms, mechanical interlocking is largely independent of surface chemistry (e.g., pH, charge, protein presence), making it particularly effective in wet or physiologically variable environments where covalent or ionic bonding might be hindered. The entanglement of polymer chains within tissue features contributes significantly to the mechanical strength and stability of the adhesive interface. This mechanism is inspired by natural adhesion strategies observed in gecko feet, octopus suckers, and worm mucus networks, where physical structures—not chemical bonds—play the dominant role in adhesion, as illustrated in Figure 7 [4,44,45,46,47,48].

### 2.3. Chemical Bonds

Chemical bonding involves the formation of covalent or ionic bonds between functional groups on the adhesive and reactive groups on tissue surfaces, such as amino, carboxyl, thiol, or hydroxyl groups [41,49,50,51]. Common chemistries used in tissue adhesives include:

- Methacrylates: often used in photopolymerizable adhesives, these groups undergo radical polymerization upon exposure to light. Although effective, their need for external initiation (e.g., UV light) can limit intraoperative utility.
- Aldehyde Chemistry: aldehydes can react with primary amines in tissues to form Schiff bases (imines), which may further stabilize via crosslinking. However, some aldehydes (e.g., glutaraldehyde) are associated with cytotoxicity and must be used with caution.
- NHS-Esters: N-hydroxysuccinimide esters react rapidly and selectively with primary amines, forming stable amide bonds. Their high reactivity and relatively low toxicity make them attractive for bioadhesive development.
- Epoxides and Isocyanates: these groups can form covalent bonds with various nucleophilic tissue groups. However, while epoxides are generally less reactive than isocyanates, both groups can pose biocompatibility challenges. Isocyanates, in particular, are highly reactive and potentially toxic, which severely limits their use in medical products. To improve biocompatibility, their application often requires chemical modifications, such as the addition of blocking groups.

The strength of chemical bonds far exceeds that of physical interactions, with bond energies ranging from 100 to 1000 kJ·mol^−1^. The strategic use of such reactive groups allows the creation of robust, durable adhesives capable of withstanding physiological stresses.

## 3. Adhesion Tests

The development and evaluation of medical adhesives require accurate and reproducible characterization of their adhesion performance. Adhesion strength must be sufficient to hold tissues together in the presence of physiological fluids, under mechanical stress, and in a dynamic environment. To this end, several standardized tests are commonly employed to assess the mechanical properties and performance of adhesives under conditions simulating those found in vivo.

### 3.1. Overview of Adhesion Testing Methods

**Figure 8:**
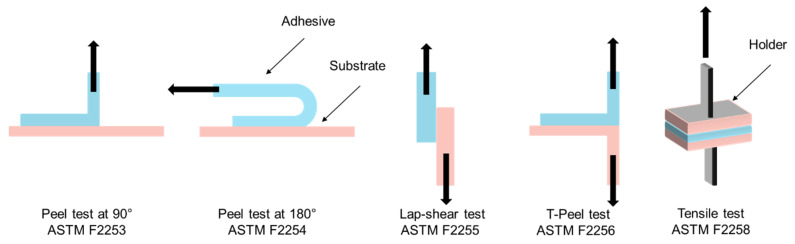
Different adhesion tests for biomedical devices/tissue adhesives with norms.

Evaluating the performance of surgical adhesives requires standardized adhesion tests that simulate the mechanical stresses encountered in vivo. These tests quantify the force necessary to separate adhered surfaces and help identify failure modes—whether interfacial, cohesive, or substrate-related. The most commonly employed tests in both the adhesive industry and surgical applications include the 90° and 180° peel tests, the lap shear test, the T-peel test, and the tensile test. Each of these methods provides valuable insights into the mechanical behavior and failure characteristics of surgical adhesives. These tests are illustrated in Figure 8 [6,52,53,54,55].

- Peel Tests (90° and 180°): the peel test measures the force required to detach an adhesive from a substrate at a specified angle, typically 90° or 180°. This method is especially relevant for adhesives applied to flexible substrates such as skin or thin membranes. In the 90° peel test, the adhesive is peeled perpendicularly from the backing, making it particularly sensitive to the cohesive strength of the adhesive. In contrast, the 180° peel test involves peeling the adhesive parallel to the substrate, more accurately replicating the forces that surgical adhesives might experience on curved or expansive body surfaces.
- Lap Shear Test: the lap shear test evaluates the shear strength of an adhesive by applying a force parallel to the bonded surfaces. This test is particularly suited for adhesives used in anatomical regions subject to lateral stresses, such as joints or internal tissues under tension. It is especially informative for characterizing gel-based polymer adhesives and viscoelastic glues, where the ability to withstand shear stress without rupturing is critical.
- T-Peel Test: the T-peel test is a variation of the peel test designed to assess the peel strength of adhesives applied to soft, deformable substrates such as skin or membranes. It provides important data on adhesive performance in dynamic environments where bonded surfaces undergo frequent movement or mechanical deformation.
- Tensile Test: the tensile test measures the resistance of an adhesive bond to a force applied perpendicularly to the bonded interface, up to the point of complete failure. This test is particularly relevant for assessing adhesives used in wound closure under tension. It provides key parameters such as tensile strength and ultimate bond strength. A cohesive failure indicates that the adhesive itself has ruptured, while an interfacial failure points to a breakdown in adhesion to the tissue.

Despite their utility, no single test can fully replicate the complex biomechanical environment of human tissues. Therefore, a combination of these methods, along with ex vivo and in vivo studies, is recommended to achieve a comprehensive performance profile. However, inconsistencies in protocols, substrates, and conditions across studies reduce comparability and reproducibility.

### 3.2. Failure Modes

**Figure 9:**
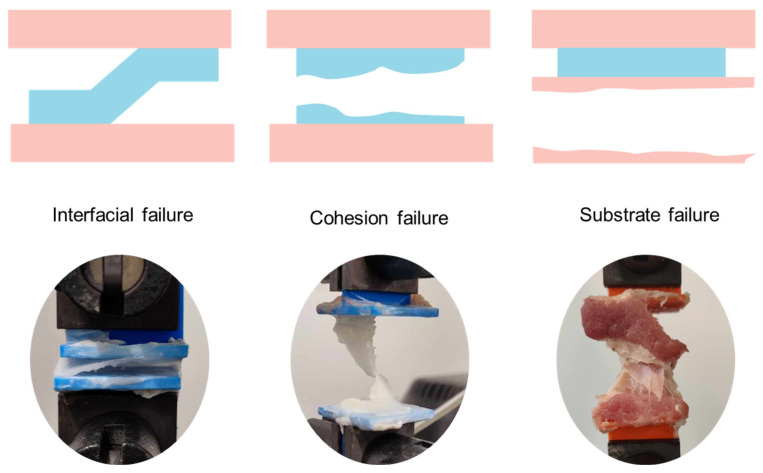
Scheme and pictures of interfacial, cohesion, and substrate failure.

In adhesion testing, the nature of the failure provides valuable insights into the performance of the adhesive system [56] (Figure 9):

- Adhesive Failure: occurs when the bond between the adhesive and the substrate fails, indicating poor interfacial interaction.
- Cohesive Failure: the failure occurs within the adhesive material itself, suggesting strong adhesion to the substrate but insufficient cohesive strength.
- Substrate Failure: the tissue or test material fails before the adhesive, indicating superior bonding strength.
- Mixed Failure: a combination of the above, typically considered acceptable if the adhesive bond is maintained under stress.

Although cohesive and mixed failures are generally preferred, the interpretation must consider the clinical context. For instance, substrate failure in fragile tissues may indicate excessive bond strength that could impair healing.

### 3.3. Parameters Affecting Adhesion Performance

Multiple factors can influence the outcome of adhesion tests and must be carefully controlled or considered when comparing materials such as substrate properties (e.g., porosity, roughness, and compliance); surface preparation and cleanliness; adhesive application method and volume; curing time and environmental conditions (temperature, humidity, and pH) and presence of fluids (e.g., blood, plasma, and moisture). Biological variability, particularly when using ex vivo tissues, also plays a role and necessitates the use of statistically significant sample sizes.

### 3.4. Regulatory Considerations

The US Food and Drug Administration (FDA), ASTM International, and ISO provide guidelines and standardized protocols for evaluating medical adhesives. Regulatory approval of a surgical adhesive requires comprehensive testing that includes mechanical performance, cytotoxicity, biodegradation, and in vivo efficacy.

However, many published studies rely on in-house protocols or limited sample sizes, with few providing long-term in vivo data or robust clinical trial outcomes. As a result, the translation of experimental results to clinical practice remains uncertain. Greater emphasis on reproducibility, compliance with regulatory standards, and transparency in experimental design is urgently needed to improve the quality and reliability of biomedical adhesive research.

## 4. Skin Pressure-Sensitive Adhesives

The primary requirement for an adhesive to bond to a substrate is a fundamental thermodynamic principle: the surface energy of the adhesive must be equal to or lower than that of the substrate—in this case, human skin. If this condition is not met, adhesion cannot occur. Additional factors influencing adhesion include the wetting rate and the viscoelastic properties of the adhesive [57].

The skin, as the largest organ in the human body, plays a key role in the immune system [58]. The outermost layer, the stratum corneum, consists of both lipophilic and hydrophilic domains made of keratin. This layer, approximately 25 µm thick, contains around 20% water. Clean, dry skin is predominantly lipophilic, resulting in a low surface energy (~25 mN·m^−1^). In contrast, moist or impure skin is more hydrophilic, with a higher surface energy (~40 mN·m^−1^ at 23 °C and 34% relative humidity, and ~60 mN·m^−1^ at 36 °C and 50% relative humidity) [57,59].

**Figure 10:**
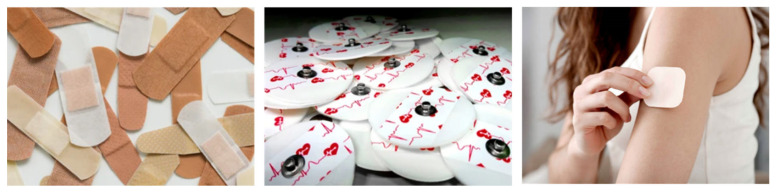
Three major markets for skin adhesives: dressings, electrodes, and transdermal patches.

Currently, most skin adhesives are based on pressure-sensitive adhesives (PSAs), which are viscoelastic polymers that bond to surfaces temporarily upon the application of pressure. The adhesion of PSAs is governed by a balance between flow and resistance to flow [60]. The largest segment of PSAs for skin is used for wound protection. Initially, hospital tapes were made from natural rubber [57], but these have since been replaced by synthetic materials. In 1899, Johnson & Johnson (J&J) introduced the first synthetic medical tape. In 1920, they developed the widely used, multi-purpose skin adhesive—plaster—by placing a piece of gauze over a strip of tape, calling it Band-Aid^®^ (J&J). Nexcare^TM^ (3M) is another well-known brand. The second largest market for medical adhesives is for electrode attachment (e.g., for electrocardiogram measurements or transcutaneous electrical nerve stimulation), with 3M’s Red-Dot^TM^ dominating this space. The third market involves Transdermal Drug Delivery Systems (TDDS) (Figure 10) [61], which, although small in terms of the number of units sold, are significant due to the high cost of devices. In all three cases, PSAs are used. These adhesives are non-reactive, operate at room temperature through substrate wetting, and lose adhesion as the temperature drops. Their adhesion depends on the viscoelasticity of the adhesive and Van der Waals interactions with the substrate. Typically, PSA formulations consist of an elastomer (e.g., polyacrylates) and a tackifier (e.g., ester rosin).

**Figure 11:**
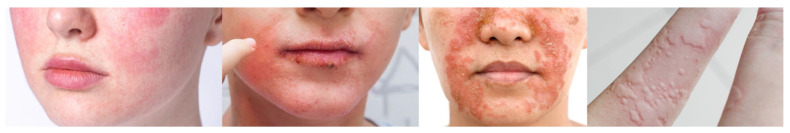
Examples of inflammatory skin conditions potentially relevant to adhesive biocompatibility studies. From left to right: mild erythema and dryness on the cheeks, suggestive of mild atopic dermatitis or rosacea; perioral inflammation and fissuring, potentially indicative of perioral dermatitis or contact dermatitis; severe facial inflammation with exudation and crusting, consistent with infected atopic dermatitis or possibly eczema herpeticum; pronounced xerosis and fissures on the fingers, potentially associated with chronic hand eczema or severe skin dryness.

For an adhesive to be suitable for dermal use, it must not cause skin reactions [52,62]. The primary skin irritations include primary irritation, assessed by checking for erythema and edema after removal, and skin sensitization, where the same test is repeated multiple times a week on the same skin area, followed by testing on different skin regions [59]. The varying levels of skin irritation are illustrated in Figure 11. It is important to distinguish that skin sensitization involves immune system activation, while skin irritation does not.

Depending on the application, the duration of adhesion can vary greatly, from a few hours for a standard bandage to several days for a TDDS (e.g., 24 h for a nicotine patch to 7 days for a contraceptive patch). Regardless of the intended duration, the adhesive must be comfortable to wear, removable without causing trauma to the skin, and without leaving residue. Despite being a thin layer, the skin is a very efficient barrier, making transdermal drug delivery a significant challenge.

**Figure 12:**
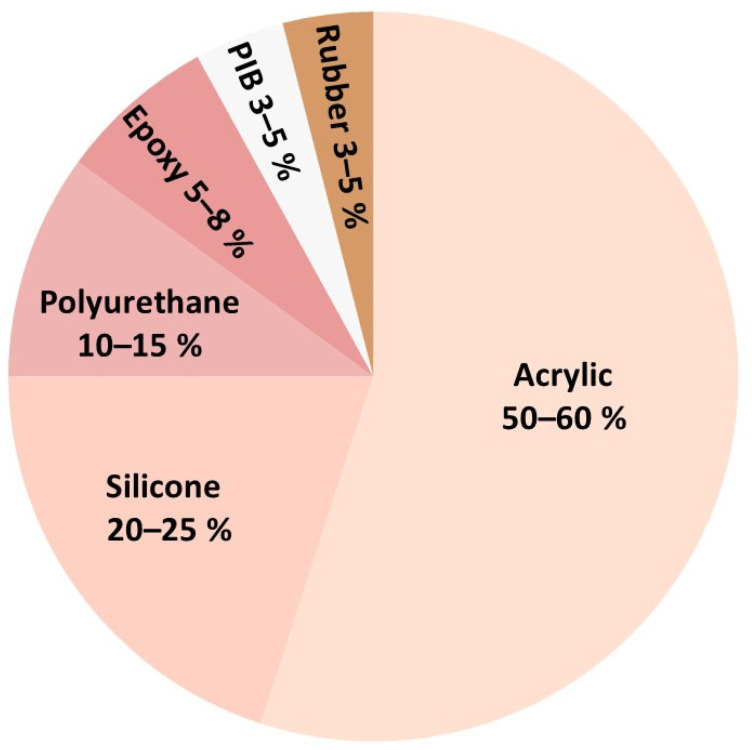
Medical pressure sensitive adhesives market by resin in 2023 [63].

The adhesive properties of medical PSAs are evaluated based on three criteria: tack, adhesion, and cohesion. Tack is defined as the initial adhesion, referring to how well the adhesive sticks to the skin with slight pressure. This phenomenon is dependent on surface wetting and increases with pressure and time. Adhesion, or peel adhesion, refers to the sum of forces at the interfaces—specifically, the force required to separate two surfaces. In the case of PSAs, adhesion is mechanical, relying on the filling of pores and electrostatic interactions (Van der Waals forces). Cohesion, or shear strength, refers to the internal attraction of molecules to one another and their ability to adhere to themselves, as defined by the American Society of Testing Materials [60]. The market for various polymers used in PSAs is illustrated in Figure 12. These polymers will be detailed in the following sections.

When selecting polymers for PSAs, their mechanical properties, particularly the glass transition temperature (Tg), are critical. Polymers with a low Tg (below room temperature) remain flexible and tacky at normal temperatures, enhancing adhesion. Examples include polyisobutylene (PIB) and styrene-butadiene copolymers, which have low Tg and contribute to a soft, tacky adhesive. To enhance adhesion, tackifiers (such as rosin or hydrocarbon resins) are often incorporated. These additives improve surface spreading and bonding, and they lower the effective Tg of the adhesive, enabling stronger adhesion even on rough surfaces. Additionally, plasticizers can be used to increase the flexibility of the adhesive, making it easier to spread and penetrate surface pores, which enhances adhesion without hardening the material. In summary, the polymers used in PSAs are selected to balance adhesion and cohesion, ensuring optimal bonding to surfaces while maintaining internal integrity. The following sections will describe the different types of polymers used in PSAs and their properties.

### 4.1. Polyacrylates

The largest group of skin adhesives is composed of polyacrylates, which have largely replaced rubber-based pressure-sensitive adhesives (PSAs) due to their superior adhesion, enhanced stability, strength, and low allergenic potential [58]. While the adhesive properties of acrylates have been recognized since 1928, they have only been used as PSAs since 1950 [64].

This polymer family is biocompatible, adheres effectively to the skin, and exhibits excellent compatibility with a wide range of drugs, making it ideal for transdermal drug delivery systems (TDDS). Their synthesis can be achieved via solvent-based, water-based, or solvent-free methods, typically through statistical copolymerization (Table 1) of monomers with homopolymers that display either high or low glass transition temperatures (Tg). Hard segments, such as vinyl acetate, contribute a high Tg (above room temperature), while soft segments, such as n-butyl acrylate or 2-ethylhexyl acrylate, result in a lower Tg (below room temperature). The resulting material attains an intermediate Tg, balancing the properties of both segments. Soft segments provide tack and adhesion, while monomers with intermediate Tg, such as methyl or ethyl acrylate, contribute internal strength. The Tg of the final adhesive typically ranges from −55 °C to −15 °C. Due to its inherent tackiness, additional tackifiers or plasticizers are unnecessary, and the adhesive functions similarly to resin/tackifier blends. Moreover, its robust stability eliminates the need for stabilizers, which could potentially cause skin irritation. Most acrylic adhesives are approved for skin contact. However, acrylic PSAs may cause mild skin irritation upon removal, which can lead to the detachment of skin cells [58]. These adhesives are commonly used in tapes, bandages, and TDDS [57,61]. Monomers with functional groups such as acids or amides can also serve as reactive sites for crosslinking [64]. Acrylic adhesives typically exhibit low to high tack and cohesion, with medium to high adhesion [60]. Their surface tension ranges from approximately 30 to 45 mN·m^−1^ [59].

### 4.2. Silicone

**Scheme 2:**
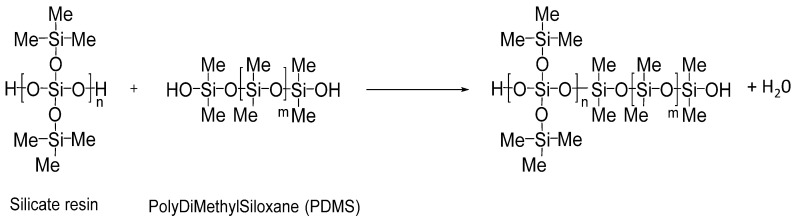
Polycondensation of silicone monomer leading to a silicone-based PSA.

Silicones, which have been used in medical devices since 1950, remained undesirable for a long time due to their high cost and initial single-source availability. However, they represent a significant family of pressure-sensitive adhesives (PSAs). Silicone PSAs are formulated based on the interaction between silicone resins (typically benzene-soluble silicate resins) and silicone polymers, particularly poly(dimethylsiloxane) (PDMS) (Scheme 2). PDMS serves as the base polymer, providing flexibility and tack, functioning as a natural tackifier. The adhesive properties depend on the ratio of resin to PDMS: increasing PDMS results in a softer, stickier adhesive, while increasing resin enhances cohesive strength and overall adhesion. This flexibility allows the adhesive to work effectively on both low-energy and high-energy surfaces, making it versatile for medical applications. Furthermore, the number of free silanol groups (Si-OH) influences bonding efficiency by improving surface interactions [64]. In contrast to polyisobutylene PSAs, which are physical blends of low and high Tg components, silicone-based PSAs are a one-component system. The Tg of these systems is −127 °C. Silicone-based PSAs are among the few adhesives suitable for the release of amine-terminated drugs. For such applications, the terminal silanols need to be end-capped. Pharmaceutical-grade silicone PSAs are commercially available (e.g., BioPSA^®^ by Dow Chemicals, Midland, TX, USA) in various tack values [61]. Silicone adhesives typically exhibit low tack, low to medium adhesion, and high cohesion [60] The surface tension of PDMS is approximately 22 mN·m^−1^ [59].

### 4.3. Polyurethanes

Polyurethanes, synthesized through the polymerization of diols or polyols with di- or poly-isocyanates, are well-known for their excellent adhesivity [61]. As a result, they are suitable for use as pressure-sensitive adhesives (PSAs), particularly due to their Tg below −30 °C for long-chain polyols. Polyurethanes typically exhibit low tack and low to medium adhesion and cohesion [60]. Being hydrophobic yet permeable to air, polyurethanes are highly valued in wound dressing applications, as they allow patients to shower while preventing infection. Although more expensive than acrylics or rubbers, polyurethanes are reliable, flexible, and durable. Their surface tension ranges between 30–50 mN·m^−1^. Polyurethane PSAs are used in various medical applications, including medical tapes and wound dressings (e.g., Tegaderm™ by 3M, Saint Paul, MN, USA), ostomy pouches (e.g., Stomadhesive^®^ by Convatec, London, UK), transdermal drug delivery systems (e.g., Nicoderm^®^ CQ, Kenvue, Canada), and TENS electrodes (e.g., Activa^®^ by Medtronic, Tolochenaz, Switzerland).

### 4.4. Polyisobutylene

**Scheme 3:**
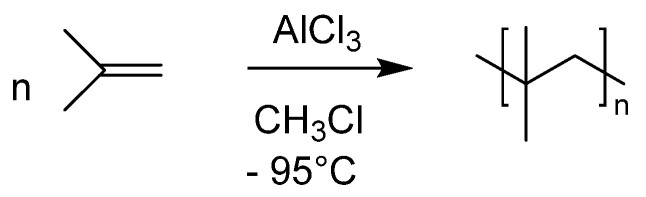
Cationic polymerization of isobutylene.

Poly(isobutylene) (PIB), first produced by BASF in 1931, is an elastomer synthesized via the cationic polymerization of isobutylene (Scheme 3). To optimize tack and adhesive performance, formulations typically use a blend of high and low molecular weight PIBs. In such systems, low molecular weight PIB acts as a tackifier, while the high molecular weight fraction serves as the primary adhesive polymer [61]. Composed solely of carbon and hydrogen, PIB is chemically inert and highly resistant to degradation. One of the earliest transdermal drug delivery systems (TDDS) was based on PIB, and it is now FDA-approved for such applications. As an amorphous polymer with a low glass transition temperature (Tg ≈ −62 °C), PIB provides excellent flexibility and maintains consistent tack over time. However, PIB-based PSAs inherently exhibit limited tack, which is why external tackifiers—such as rosin esters—are often added. These tackifiers enhance the polarity of the formulation, especially when used alongside plasticizers (e.g., mineral oils, phthalates), fillers (e.g., silicas), waxes, or other additives. Rosin esters, commonly derived from pine, are preferred for their low toxicity, though residual acids can cause irritation or sensitization in some cases. Thanks to PIB’s intrinsic stability, stabilizers are generally unnecessary. Since ready-to-use PIB-based PSAs are not commercially available, manufacturers must formulate their own blends using available low and high molecular weight PIBs (e.g., Oppanol^®^ by BASF, Ludwigshafen, Germany, Vistanex^®^ by Exxon Chemical, Leatherhead, UK) and, if needed, tackifying resins such as Escorez^®^ (Exxon Chemical, Leatherhead, UK). The surface tension of PIB is approximately 31 mN·m^−1^ [59].

### 4.5. Rubber

Natural rubber, composed of polyisoprene extracted from *Hevea brasiliensis*, was the first polymer used to produce pressure-sensitive adhesives (PSAs). In 1845, Horace Day applied natural rubber onto a strip of cloth to create the first medical adhesive tape—marking the birth of PSA technology. Today, natural rubber has largely been replaced by synthetic rubbers, primarily copolymers of butadiene, styrene, and ethylene (in block or random configurations), due to the high cost and allergenic potential of natural rubber. In both natural and synthetic rubber-based PSAs, the base polymer is formulated with various additives—tackifying resins, plasticizers, antioxidants, oils, and fillers—to enhance performance and reduce costs. Rubber alone does not provide sufficient tack or adhesion, so these additives are essential. These formulations remain among the most cost-effective options for medical adhesives. However, synthetic rubber PSAs tend to exhibit modest adhesive performance that degrades over time. Even at the start of their service life, their adhesion properties are generally lower than those of acrylic- or silicone-based PSAs. Synthetic rubbers typically show high tack and medium to high adhesion and cohesion [60]. Their surface tension, around 30–35 mN·m^−1^, is higher than that of dry skin (~25 mN·m^−1^) [59], which limits adhesion according to thermodynamic principles. Furthermore, natural rubber latex contains allergenic proteins, making it a common source of skin allergies, which has contributed to its decline in medical applications.

### 4.6. Smart Adhesives

Stimuli responsive adhesives are promising materials for wound care [61,62,63,64,65]. These ‘smart adhesives’ can reversibly change their adhesive properties (such as tack, cohesion or debonding behavior) in response to various triggers, including physical pressure (PSA) [66], pH [67,68,69], temperature [70,71,72], light [73,74,75], or even enzymes [76]. These smart adhesives enable controlled adhesion and detachment, making them particularly suitable for biomedical applications, including wound dressings, wearable sensors and surgical tapes. Inspired by nature, many of these new materials also incorporate substances such as antimicrobial agents or growth factors to promote faster healing [77].

One notable example of a pressure-sensitive material is a novel shear-stiffening adhesive composed of poly(diborosiloxane)/SiO_2_ (sPDBS) [66]. This adhesive not only modulates its adhesion strength in response to the applied mechanical force but also exhibits antimicrobial properties. In a comparative study, Huang et al. benchmarked the performance of sPDBS against conventional commercial adhesives, including acrylic and silicone-based formulations. Their findings revealed that, due to the material’s dynamic bonding capacity and shear-stiffening characteristics, sPDBS exhibited enhanced, rate-dependent adhesion. This performance surpasses that of traditional adhesives, indicating the potential of sPDBS as a multifunctional and efficient alternative in applications requiring tunable adhesive properties. Moreover, pH-responsive adhesives exploit the elevated pH of infected tissues (7–9 vs. 4–6 in healthy skin) [78] to modulate adhesion. Materials such as poly(acrylic acid) reduce adhesion at high pH due to chain repulsion, while chitosan enhances adhesion in acidic environments through electrostatic interactions [79,80,81]. Cintron-Cruz et al. designed a tissue adhesive with alginate, polyacrylamide, and chitosan, achieving up to 3200 J·m^−2^ at pH 9, demonstrating how pH sensitivity can optimize adhesion in inflamed or infected areas [79]. Thermo-responsive pressure-sensitive adhesives (PSAs) exploit transitions in polymer properties, such as the glass transition temperature (Tg) or lower critical solution temperature (LCST), to modulate viscoelasticity and adhesion. For example, poly(N-isopropylacrylamide) (pNIPAM) loses adhesiveness above its LCST due to hydrogel dehydration and shrinkage. Thermoplastic elastomers such as styrene-isoprene-styrene (SIS) can also be tuned to adhere at body temperature and detach at lower temperatures [82]. Zhang et al. developed a hyaluronate/gelatin-based hydrogel incorporating Rhein and silver, showing improved adhesive (up to 12 kPa) and antibacterial performance, with accelerated wound healing and reduced blood loss [83]. The thermo-responsive behavior of gelatin allowed strong tissue adhesion at 37 °C and painless removal upon cooling. Similarly, Jiang et al. formulated a pNIPAM-based hydrogel with silver nanowires and found it promoted healing while significantly reducing bacterial viability, with temperature-dependent conductivity (3.5% °C^−1^), highlighting its multifunctional therapeutic potential [22].

Light-responsive adhesives, particularly those activated by UV or near-infrared (NIR) light, offer non-invasive control over adhesion and therapeutic functions [84]. Molecules such as azobenzene and coumarin can undergo structural changes upon light exposure, enabling reversible adhesion. Shi et al. developed a dual-responsive hemostatic hydrogel utilizing cellulose nanofibrils (white light-activated antibacterial action) and Prussian blue nanoparticles (NIR-induced softening for painless removal) [85]. Similarly, Huang et al. designed a silk fibroin-based hemostat incorporating chlorin e6, achieving >90% bacterial reduction and significantly reduced blood loss following UV and NIR exposure [86].

Hydrogel-based adhesives are ideal for biomedical applications due to their biocompatibility, biodegradability, and moisture absorption [63]. These materials support healing, offer strong adhesion, and maintain oxygen permeability while preventing bacterial growth [87]. Smart hydrogels can also respond to physiological stimuli (e.g., pH, temperature, and glucose) and are being integrated with microelectronics for real-time wound monitoring [61,62,77,88]. For instance, Jiang et al. equipped their adhesive hydrogel with a Bluetooth module to wirelessly transmit temperature data, enabling early infection detection [82]. Despite these promising advances, significant challenges remain. These include ensuring consistent and reliable power sources for embedded electronics, developing environmentally safe disposal methods for integrated systems, and achieving seamless integration with existing commercial wound care products. Addressing these limitations will be key to translating smart adhesive technologies into widespread clinical use.

## 5. Surgical Use Adhesives

Surgical adhesives are increasingly recognized as valuable alternatives or complements to sutures and staples, offering benefits such as reduced operative time, minimized scarring, and improved patient comfort. However, their clinical effectiveness is often limited by a mismatch between adhesive properties and the diverse mechanical, chemical, and biological demands of different tissues. For instance, vascular tissues require adhesives that can withstand pulsatile flow, lungs demand elasticity for expansion and contraction, and the dura mater necessitates watertight sealing with minimal swelling to avoid neural compression [89].

Despite these distinct requirements, many adhesives are still developed with a generalized “one-size-fits-all” approach. This often results in suboptimal performance. Fibrin-based adhesives, while biocompatible and widely used, suffer from poor mechanical strength and risk of pathogen transmission. Excessive use of fibrin glues may compromise wound healing and effective remodeling of the adhesive. Cyanoacrylates offer rapid bonding but are associated with tissue toxicity and poor performance in moist environments [90]. They have been reported to cause inflammation, tissue necrosis, and thrombotic events when used clinically. Their degradation releases formaldehyde and cyanoacetate, contributing to cytotoxicity and potential mutagenicity. Short-chain variants degrade faster and release more toxic byproducts. Although medical-grade formulations are less toxic, concerns remain about their long-term safety and metabolic fate [91]. Albumin-glutaraldehyde adhesives such as BioGlue provide strong support but raise concerns about inflammation and slow degradation. BioGlue offers structural support in cardiovascular surgery but poses significant clinical risks, particularly due to its potential to cause swelling and mass effect. Its expansion can compress nearby structures, leading to complications such as spinal cord compression, pseudoaneurysm, or valve dysfunction. Spillage or leakage may result in embolization, causing ischemic events. To minimize these risks, meticulous application techniques and protective measures are essential during use [92]. PEG-based hydrogels are flexible but prone to excessive swelling, while gelatin- and polyurethane-based adhesives may lack durability or degrade too slowly. PEG-based surgical adhesives, such as DuraSeal^®^ and Adherus^®^, are indeed widely used in neurosurgery for achieving watertight dural closure. However, their hydrophilic nature can lead to significant post-application swelling—up to 46% in volume—which poses a serious risk in confined anatomical spaces. Clinical reports have linked DuraSeal^®^ to complications such as cauda equina syndrome and spinal cord compression due to this expansion. In contrast, Adherus^®^ exhibits reduced swelling and comparable clinical effectiveness, potentially offering a safer alternative in sensitive neurological procedures [93]. Polysaccharide-based adhesives such as chitosan and dextran face solubility and biodegradability challenges [89,90].

These limitations highlight the need for next-generation adhesives that are tissue-specific, mechanically robust, and biologically safe. Future adhesives should offer controlled degradation, maintain adhesion in wet or inflamed environments, and be compatible with minimally invasive techniques. Innovations such as photo-crosslinkable systems, mussel-inspired chemistries, and multifunctional platforms integrating drug delivery or bioelectronics are promising directions [93].

Ultimately, the future of surgical adhesives lies in precision design—where materials are engineered to meet the specific demands of each tissue and surgical context, ensuring safer, more effective, and more versatile clinical outcomes [89,90,94].

### 5.1. Wound Closure Adhesives

Also known as topical surgical glues, wound closure adhesives are liquid formulations that polymerize, solidify, or gel upon application, forming a flexible film that holds wound edges together and promotes healing without the need for invasive staples or sutures. These adhesives are fast-acting and easy to apply. Most commonly, they are based on cyanoacrylates (e.g., Dermabond^®^, Ethicon, Raritan, NJ, USA), but polyurethane-based formulations also exist (e.g., Cutis, Adhesys Medical, Aachen, Germany). These products serve not only as adhesives but also as sealants, creating a protective barrier that reduces the risk of infection and prevents fluid leakage. An additional benefit is their potential to reduce scarring. In the case of liquid adhesives, adhesion may occur through chemical interactions such as ionic or covalent bonding between the adhesive and tissue [59].

### 5.2. Implantable Surgical Adhesives

Implantable surgical adhesives are designed for internal use and play a crucial role in advanced surgical procedures. They can be employed to affix implantable devices such as prostheses, or to ensure airtight and watertight seals in delicate surgeries involving organs such as the lungs or blood vessels. These adhesives must be highly biocompatible, degrade safely without producing toxic byproducts, and avoid triggering inflammatory or immune responses.

#### 5.2.1. Biological and Biochemical Adhesives

The term “biological” refers to adhesives derived from organic, renewable sources—primarily plants and animals. Natural polymers such as polysaccharides and proteins inherently possess adhesive properties, making them valuable in both industrial and medical applications [95,96]. In medicine, these biomaterials serve a wide range of purposes, from tissue adhesives to scaffolds for regeneration. Their adhesion typically relies on biochemical crosslinking reactions. These materials are often sourced from animal tissues, such as bovine, porcine, or crustacean origins. They are generally non-toxic, biocompatible, physiologically degradable, and possess hemostatic properties, making them suitable for internal use. Many of these adhesives are commercially available, and several have been approved by the US FDA. One of the main advantages of these natural adhesives is their excellent biocompatibility; they typically do not provoke immune reactions, and their degradation products are safe for the body. Some also offer intrinsic antimicrobial properties [24]. However, challenges remain, such as batch-to-batch variability and the potential risk of disease transmission from animal-derived components [2]. Among natural materials, protein-based adhesives are particularly noteworthy for their ability to closely mimic physiological mechanisms, offering a high degree of compatibility with human tissue and healing processes.

##### A. Based on Proteins

Proteins derived from natural sources are extensively employed in the formulation of surgical adhesives [12,97,98,99]. These proteins may be extracted from human blood or isolated from animal tissues, typically of bovine or porcine origin. Depending on their composition and intended application, protein-based adhesives can function either without additional chemical reagents—as is the case with fibrin glues—or in combination with reactive compounds such as aldehydes or N-hydroxysuccinimide (NHS) esters to enhance bonding strength. This category primarily includes fibrin-, gelatin-, and albumin-based adhesives, each offering distinct properties and clinical benefits. Their natural origin contributes to their excellent biocompatibility, and many of these adhesives are already in widespread clinical use. Table 2 provides an overview of the key characteristics of protein-based surgical adhesives. It categorizes the products by their primary function (adhesive, sealant, or hemostat), outlines the underlying technology, indicates the date of first surgical use, and includes information on adhesion strength, cost, regulatory approvals (US FDA and/or CE marking), storage and application conditions, degradation time within the body, and examples of commercial products.

(a) Fibrin

**Figure 13:**
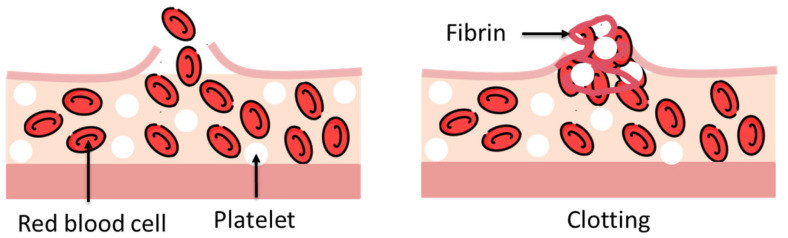
Blood clotting in the presence of fibrin.

A large number of biocompatible adhesives are inspired by the body’s natural coagulation cascade—fibrin glue being a prime example. Fibrin itself is an insoluble protein that does not exist in a free form in the body but is produced in situ through the enzymatic reaction between thrombin and fibrinogen. Fibrinogen is a soluble plasma protein, while thrombin is a key enzyme involved in the clotting process. When combined, these two components trigger the final stage of blood coagulation, forming an insoluble fibrin clot. As illustrated in Figure 13 fibrin forms a mesh-like network that entraps blood cells, effectively mimicking the natural hemostatic process [4,101].

**Figure 14:**
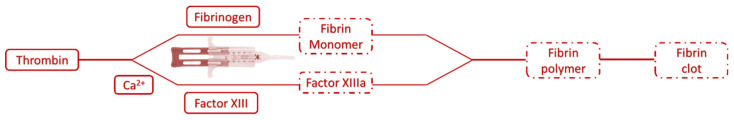
Mechanism of polymerization of fibrin glue [102].

This type of adhesive is formulated as a two-component system: thrombin on one side, and fibrinogen, calcium ions (Ca^2+^), and fibrinase (Factor XIII) on the other. The mechanism of action is illustrated in Figure 14. Thrombin acts on fibrinogen to produce fibrin monomers, while simultaneously reacting with calcium ions and Factor XIII to generate the active enzyme Factor XIIIa. When both components are mixed, Factor XIIIa catalyzes the crosslinking of fibrin monomers into polymeric fibrin, ultimately forming a stable blood clot.

Its polymerization process is heat-free, allowing for the growth of neo-tissues. This pseudo-synthetic clot degrades similarly to the naturally formed clot, with its degradation occurring via thrombolysis over a period ranging from a few days to several weeks. In some instances, an antifibrinolytic agent, such as aprotinin, is incorporated to modulate the gelation kinetics and prevent premature lysis [2,4]. Both its production and degradation are residue-free, ensuring that fibrin glues are entirely biodegradable and bioresorbable, without inducing immune responses. However, as fibrinogen is sourced from humans and thrombin is derived from pooled human plasma or bovine blood, there remains a significant risk of bloodborne pathogen transmission, including HIV, Hepatitis B and C, and even prion diseases.

The tissue adhesive properties of fibrin were first documented in 1940 [103]. At that time, purified thrombin was combined with concentrated plasma for use as an adhesive in skin grafting. Despite its early promise, its mechanical properties were initially insufficient, preventing widespread adoption. The adhesive strength of fibrin is closely linked to the concentration of available fibrinogen. Cryoprecipitation of blood remains the gold standard for fibrinogen extraction [104]. Fibrin glue began to see clinical use in Europe when highly concentrated fibrinogen became available [105]. Since then, fibrin glue has emerged as one of the most commonly used biobased surgical adhesives [3,4,22,106]. It is widely employed for primary wound closure, skin grafts, and in a variety of surgeries, including orthopedic, periodontal, plastic, reconstructive, ophthalmic, urological, and laparoscopic procedures. Additionally, it is used in conjunction with sutures and staples, where its primary role is to prevent bleeding and fluid leakage during and after surgery, such as in lung surgery, neurosurgery (e.g., dural repairs), laparoscopic surgery, and in cardiovascular and gastrointestinal surgeries.

The main drawbacks of fibrin glues include their high cost and low adhesive strength in wet environments. Additionally, the application process is restrictive: the glue must be stored frozen and should be used within 12 h after thawing if kept at 37 °C, or within 72 h if stored at room temperature. Refreezing or re-refrigerating the glue is not permitted. Despite fibrin’s advantages, such as its biocompatibility, full resorbability, and ability to support cell growth, the risks of anaphylaxis and antibody formation must be carefully considered [107]. Furthermore, its low viscosity (<100 mPa·s) limits its functionality, though it remains an effective hemostatic agent [108].

To address the risks of bloodborne pathogen transmission, automated medical devices have been developed that enable the preparation of autologous fibrin sealant from the patient’s own blood (e.g., CoStasis by Stryker, using bovine collagen, thrombin, and the patient’s plasma). However, this technology results in fibrin with a lower fibrinogen concentration, thereby decreasing its adhesive strength. Moreover, the production process for this type of fibrin takes two days, making it unsuitable for emergency surgeries or immediate wound management [2,23].

Products containing only thrombin (topical thrombin) are also available, such as Thrombin-JMI (King Pharmaceuticals, Bristol, VA, USA) and Evithrom (Johnson & Johnson, New Brunswick, NJ, USA). Similar to fibrin glue, they promote clot formation by converting fibrinogen into fibrin. These products can be used in conjunction with gelatin sponges. Bovine thrombin can be stored at room temperature (25 °C), while human thrombin requires more stringent storage conditions: at −18 °C for up to two years, at 2–8 °C for one month, at room temperature for one day, and at 37 °C for only one hour.

Studies on improving fibrin glue are limited. In 2020, Mohammadi and his team [100] introduced a nanocomposite adhesive based on PCL/fibrin. They fabricated PCL scaffolds via electrospinning and incorporated fibrin into them. The fibrin-loaded scaffolds exhibited improved hydrophilicity (with a contact angle of 81° compared to 125° for pure PCL), which enhanced both adhesion and cell growth. The loaded scaffolds also supported better cell viability. Although the exact adhesive strength was not provided, the authors noted an increase in adhesivity with the presence of fibrin compared to pure PCL. The mechanical properties of the scaffolds’ fibers were also assessed, showing a maximum stress of 6.6 MPa, a maximum strain of 60%, an elastic modulus of 10.03 MPa, and a failure strain of 85% for fibers with a diameter of 870 nm. These values were superior to those reported in the literature [109,110], attributed to fiber entanglement and the interlocking of fibrin with the PCL network.

Around the same time, Ahmed et al [111]. developed an affordable fibrin glue using the patient’s own plasma and calcium chloride. Their formulation exhibited gelation times of 3 min with fresh plasma and up to 25 min with frozen plasma. The glue demonstrated adhesion strength of 40–55 g·cm^−2^, although reduced adhesion on wet tissues was observed for short periods. The addition of 0.5% cellulose improved adhesion strength to 75 g·cm^−2^, surpassing some values found in the literature. In 2023 [112], the same authors further improved the adhesive strength by adding sodium carboxymethylcellulose (CMC) or methylcellulose (MC) to the formula. These modifications resulted in adhesion strengths of 81 g·cm^−2^ and 106 g·cm^−2^, respectively. While the glue prepared with fibrin and 10% calcium chloride formed a clot after 12 min, the formulations with CMC or MC formed clots in just 4 min. However, the CMC-based formulation showed lower cell viability, falling below the required 70% when 1.25% or more CMC, or 2.50% MC, was added.

In summary, fibrin glues, based on thrombin and fibrinogen, are FDA-approved for use as sealants, hemostats, and adhesives, mimicking the final step of physiological coagulation. While these glues are biocompatible and physiologically degradable, their animal-based origins pose a risk of transmitting blood-borne diseases. To mitigate this, autologous fibrin glues have been developed. However, due to their low adhesion strength, their applications are primarily limited to low-pressure bleeding. Additionally, their complex production process results in relatively high costs. Despite limited research on improving fibrin glue, its risk-to-benefit ratio remains favorable, making it the most widely used surgical adhesive.

(b) Collagen and gelatin

Collagen and gelatin are hemostatic agents derived from animals (typically cows and pigs). They function primarily as mechanical barriers to accelerate blood clotting, rather than directly participating in the coagulation cascade such as thrombin or fibrin. These materials are safe, easy to use, and biocompatible, but they must be removed after hemostasis is achieved to minimize the risk of infection [3]. Both collagen and gelatin are biodegradable and do not induce foreign body reactions. Additionally, they support cell growth and adhesion. Collagen is the most abundant structural protein in the animal kingdom, found in tissues such as skin, bone, cartilage, and muscle. It is a biopolymer composed of polypeptides arranged in a triple helix structure. Its primary role is to provide mechanical strength and stretch resistance to tissues, playing a vital role in wound healing. Collagen presents a lower risk of disease transmission compared to fibrin. The FDA has approved various collagen products derived from animal sources, such as bovine bones. These products are available in the form of sponges and powders. Collagen-based hemostats are typically used when other hemostatic agents are ineffective, providing hemostasis within one to five minutes, and they are absorbed by the body within two months. However, they cannot be used for wound closure as they may delay healing, nor should they be combined with cyanoacrylate glues, as they reduce the adhesive strength. Unlike fibrin, collagen products can be stored at room temperature (15–30 °C) and are more affordable, typically ranging from $50–150 for a sponge (e.g., Helistat^®^—Integra, Princeton, NJ, USA) or $30–200 for a few grams of powder (e.g., CollaStat—Dalim Tissen, Seoul, Republic of Korea, or Lyostypt^®^—Bbraun, Saint-Cloud, France) [23]. These hemostats are used in various surgeries, including cardiac procedures. However, collagen-based hemostats tend to swell, so they should be avoided in ophthalmic and urological surgeries [4].

**Figure 15:**
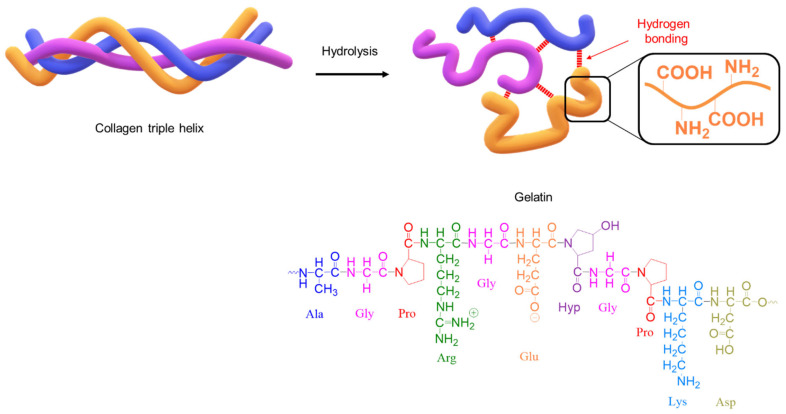
Collagen triple helix, its hydrolysis product; gelatin and backbone of gelatin.

Gelatin, a mixture of proteins and polypeptides, is derived from the partial irreversible hydrolysis of collagen (see Figure 15). This process results in a 3D network held together by hydrogen bonds between the amino acids. Gelatin is characterized by low antigenicity, supports cell growth, and is both cost-effective and readily available. Additionally, it exhibits elastic properties and can be easily modified. Due to these attributes, gelatin has been used as a hemostatic agent for centuries and was one of the first polymers used in medical adhesives. It is biocompatible, physiologically resorbable, and non-immunogenic, with peptide sequences that facilitate cell adhesion [102].

**Figure 16:**
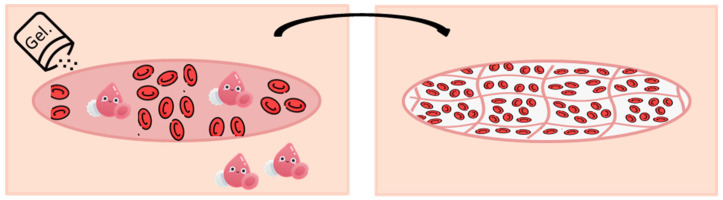
Swelling of the gelatin powder in the presence of blood, creating a physical barrier.

Gelatin works by swelling when in contact with blood, forming a clot. As illustrated in Figure 16, protein and peptide entanglements in the presence of blood result in the formation of a gel. On the downside, gelatin degrades rapidly in the body due to hydrolysis and enzymatic breakdown [113]. Gelatin-based hemostatic products are available in the form of sponges and powders, both of which can be stored at room temperature (25 °C). The cost ranges from $30–150 for a sponge and $40–120 per gram for powder, such as Surgicel^®^ (±powder) by Ethicon, Raritan, USA or Gelfoam^®^ (±powder) by Pfizer, New York, NY, USA. To enhance adhesive strength, a gelatin-based sealant called gelatin-resorcinol-formaldehyde (GRF) was developed [2,4,24]. This formulation creates a three-dimensional network through the reaction of resorcinol and aldehydes, which generate alcohol-free functional groups. The amine groups of gelatins (specifically lysine residues) and the aldehydes form covalent bonds via polycondensation with formaldehyde. GRF is used in thoracic aortic dissection and for hemostasis. However, due to the toxicity of aldehydes, this formulation was not an ideal replacement for the earlier gelatin-based materials. Formaldehyde can cause severe histopathological changes, limiting the use of GRF.

A second-generation formulation of GRF, known as GRFG, was later developed. This adhesive consists of two components that react within minutes upon mixing. One component contains gelatin and resorcinol, while the other comprises formaldehyde and glutaraldehyde, which act as crosslinking agents (e.g., GRF Biological Glue—Microval, Saint Just Malmont, France, no longer commercially available) [52]. GRFG offers improved adhesive strength—comparable to that of cyanoacrylates—and demonstrates lower cytotoxicity than traditional fibrin glues. However, its major drawback lies in the toxicity of the aldehydes used. Both formaldehyde and glutaraldehyde are known to induce inflammation, tissue necrosis, and are suspected carcinogens. Despite these safety concerns, GRFG continues to be used in specific surgical contexts, such as vascular, gastrointestinal, and pulmonary procedures. Another gelatin-based sealant combines gelatin with thrombin to enhance hemostatic performance. In this formulation, thrombin not only contributes to physical clot formation but also actively promotes coagulation. Commercially available as a matrix within a prefilled syringe (e.g., Floseal—Baxter, Glenview, IL, USA), this product is used in wound closure, bone void filling, and internal surgeries. While standalone gelatin sponges and powders are relatively inexpensive, their combination with thrombin significantly increases the cost. Because the thrombin used in these formulations often originates from bovine sources, there is a risk of disease transmission. As such, the FDA recommends strict quality controls, source verification, limited use in essential clinical applications, and careful postoperative monitoring. Furthermore, foreign body reactions have been reported, prompting recommendations to avoid use in infected sites, to limit quantities applied, and to ensure the material is removed as soon as hemostasis is achieved [2,23,28].

**Scheme 4:**
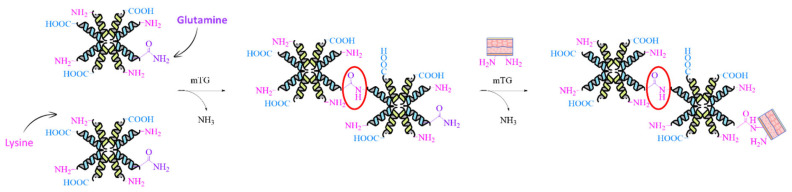
Reaction of lysine and glutamines of the gelatin, mediated by the transglutaminase.

An alternative to these sealants relies on the crosslinking of gelatin by microbial tranglutaminase catalysis (mTG). The reaction involves the glutamine of the gelatin, which reacts with amines due to the catalysis of transglutaminase. Glutamine also reacts with the amine of the body, leading to adhesion. As shown in Scheme 4, this reaction generates ammonia. Nevertheless, since NH_3_ is produced in low quantities, it can be processed by the body (converted as urea by liver, and eliminated through the kidneys, for instance) without inducing side-effects [114]. This glue received the CE mark for use in gastrointestinal surgery (LifeSeal™—LifeBond, Caesarea, Israel) and is still the subject of clinical trials in USA.

**Scheme 5:**
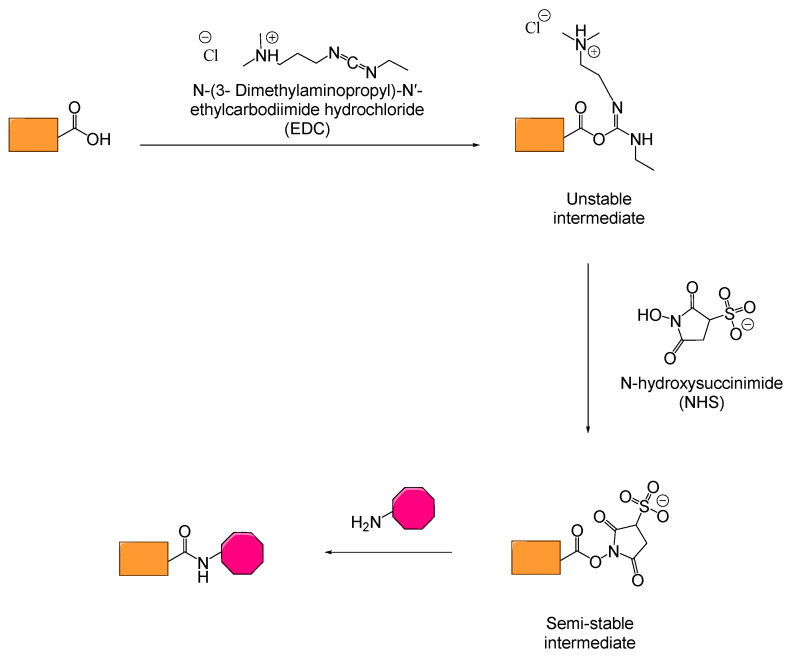
Mechanism of the addition of an amine to an acid with EDC/NHS-ester chemistry.

For decades, gelatin has been considered one of the most promising biopolymers. On the other hand, it suffers from low adhesion and poor mechanical properties [115]. In this regard, scientists have used chemical modifications (thiolation [113], methacrylation [50,116,117,118], catechol functionalization [119,120,121], etc.) or blending of gelatin [122,123]. In the past five years, the most reported modifications were methacrylation, catechol functionalization, aldehyde chemistry [16,124,125,126] and EDC/NHS [127,128] (N-(3-Dimethylaminopropyl)-N′-ethylcarbodiimide hydrochloride; EDC, N-hydroxysuccinimide; NHS; Scheme 5). To achieve the best adhesive strength, some teams also combined these chemistries [129,130,131]. In some studies, gelatin was also used in combination with other biological molecules such as chitosan [127,132], collagen [120,123], alginate [122,133], or dextran [125]. Gelatin has also been associated with dopamine in order to take advantage of the catechol chemistry relying on imide bond formation. To this end, the carboxylic groups of gelatin were reacted with the amine of the dopamine catechol. The catechol groups of the dopamine could thus be coordinated by Fe^3+^ ions, leading to the gelation of the system. A glue with a tensile strength of 20 kPa was thus formed [121]. Moreover, the two hydroxyl groups of the catechol can also be oxidized into ketones in order to react with the amines of the body to form imine bonds. The adhesion strength increases with the amount of dopamine and can reach 45 kPa in such cases [119]. When mixed with gelatin-methacrylates, dopamine-modified gelatin can achieve 150 kPa [130], but caution must be taken, as methacrylate can lead to a severe decrease of cellular viability (below the required 7%), making the formulation non-biocompatible. In addition, UV light can damage tissues and cells; in this context, several teams used visible light to induce the radical polymerization of the methacrylates [113,116,123]. In this context, fillers can be a good option to enhance adhesion. For instance, laponite [113], kaolin [122] or mesoporous bioactive glass [123,124] can increase adhesion strength of gelatin up to 110 kPa. In the meantime, they improve biocompatibility due to their antibacterial effect. These research efforts thus pave the way for non-toxic gelatin-based high strength tissue adhesives.

(c) Albumin

**Scheme 6:**
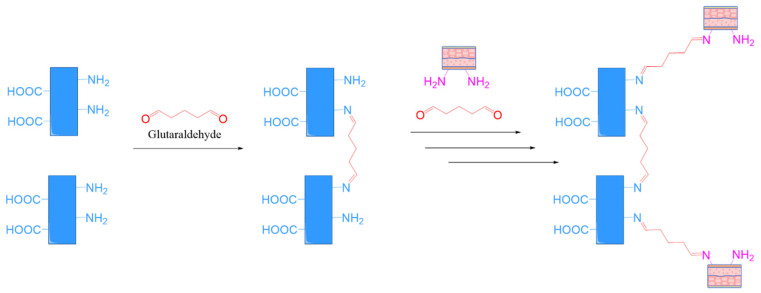
Mechanism of crosslinking of albumin with the amines of the tissues, in the presence of glutaraldehyde.

Albumin is a water-soluble protein found in blood plasma (the most abundant protein in plasma, 50–60%), made of thousands of amino acids. It can coagulate in the presence of heat, alcohol, mineral acids, etc. For surgical glue, albumin is formulated with glutaraldehyde. Human Serum Albumin (HSA), derived from human plasma, is primarily used in medical products due to its better safety profile. In contrast, Bovine Serum Albumin (BSA), although cheaper, is generally avoided in clinical formulations approved for human use due to the risk of disease transmission (e.g., bovine spongiform encephalopathy—BSE) [134,135,136]. Although glutaraldehyde is known to be cytotoxic, it is used in small amounts in surgical glues. In critical clinical situations, its effectiveness has tipped the balance clearly in favor of its benefits over potential risks. Moreover, clinical studies have shown that this type of glue does not lead to serious side effects, which has contributed to its long-standing use in vascular and cardiac surgeries. Similar to all protein-based surgical glues, albumin-based adhesives exhibit intrinsic hemostatic properties that can support the coagulation cascade. Commercial albumin glues such as NE’X Glue^®^ (Grena^®^) and BioGlue^®^ (CryoLife, Sarasota, FL, USA) are composed of a mixture of approximately 45% albumin and 10% glutaraldehyde, stored in dual-chamber systems [102]. Upon application, gelation occurs in about 30 s, with maximum adhesion achieved within two minutes. As illustrated in Scheme 6, similarly to GRFG, glutaraldehyde covalently crosslinks both the albumin and tissue proteins, creating a robust mechanical seal that acts independently of physiological coagulation. Aldehydes exhibit high reactivity due to the electronegativity difference between the carbon and oxygen atoms in the carbonyl group. When amines react with aldehydes, a Schiff base reaction leads to imine bond formation with water release. In addition, thiols may form hemi-thioacetals. This chemical process results in the rapid formation of a hydrogel within seconds to minutes. The adhesion strength of these glues is superior to that of gelatin-based adhesives and increases with higher glutaraldehyde concentrations. They also exhibit excellent tensile and shear strength. However, the formulation’s low viscosity makes precise application challenging. It must be applied to dry surgical sites and degrades slowly through proteolysis, which may trigger inflammatory responses. Moreover, if bovine-derived albumin is used, there is a potential risk of infectious agents [23,52]. Although rare, some cases of tissue edema and necrosis have been reported. Despite these concerns, BioGlue^®^ has received FDA approval for use in aortic dissection and vascular surgery. To overcome aldehyde-related limitations, Baxter has introduced a polyaldehyde-based alternative (PreveLeak), which has shown no reported adverse effects. Nevertheless, glutaraldehyde continues to be widely used due to its strong reactivity, bifunctionality, and high adhesive strength, despite its known pro-inflammatory potential.

In 1994, an alternative albumin-based sealant was introduced: PEGylated Human Serum Albumin (HSA-PEG), consisting of albumin and polyethylene glycol terminated with N-hydroxysuccinimide (PEG-NHS) [137]. Progel™ (Davol) is the only FDA-approved sealant specifically designed for lung surgery to prevent air and gas leaks [138]. It forms a hydrogel upon application through rapid crosslinking at the tissue surface. The negatively charged albumin structure enables this crosslinking within 15–30 s. Although its viscosity is low, application is facilitated by a spray mechanism, avoiding handling issues. This aldehyde-free formulation is user-friendly, sufficiently robust, and compatible with mechanical closure techniques such as sutures or staples. Since albumin-based glues are sensitive to enzymatic degradation, this formulation is naturally eliminated within less than a week. However, these products are relatively expensive and, similar to fibrin-based glues, must be stored under refrigerated conditions [2,23,24].

**Scheme 7:**
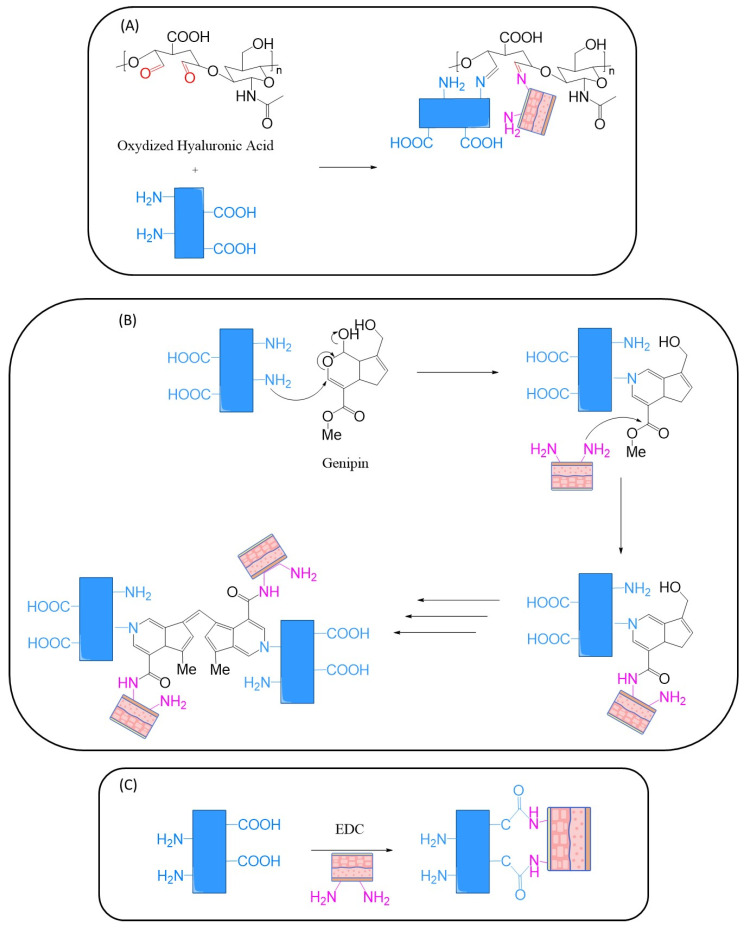
Different technologies of albumin-base glue with oxidized hyaluronic acid [139] (**A**) Genipin [140] (**B**) and EDC [141] (**C**).

In the recent years, there have not been many scientists who took interest in the design of surgical glue based on albumin. As for the albumin/glutaraldehyde glue, the described formulations are simple, based on albumin and a crosslinking agent, in order to bond covalently to the amines in the body, their mechanisms of action are depicted in Scheme 7. Liu et al. [139], synthesized a glue based on albumin/aldehyde chemistry but replaced glutaraldehyde by oxidized hyaluronic acid. They then compared it with cyanoacrylate and with AGG. They obtained a similar adhesion strength to cyanoacrylate (60 kPa) but lower than that with glutaraldehyde (115 kPa). In addition, the glue was more expensive and cured more slowly than cyanoacrylate and the glutaraldehyde-based glue. On their side, Sun et al. [140], utilized genipin as crosslinking agent. Borzacchiello and his team [111] also compared the adhesion of their formulation to albumin/glutaraldehyde glue. With a hydrogel containing 25% of aldehyde and 0.2 mol equivalent of EDC, they obtained adhesion of 90 kPa. The best adhesion they achieved with glutaraldehyde was 50 kPa with 15% or 25% of albumin and 0.05 mol equivalent of glutaraldehyde. Additionally, in contrast to glutaraldehyde, EDC does not remain a part of the glue, and is metabolized by the body. All these adhesives exhibit good biocompatibility.

##### B. Based on Polysaccharides

**Figure 17:**
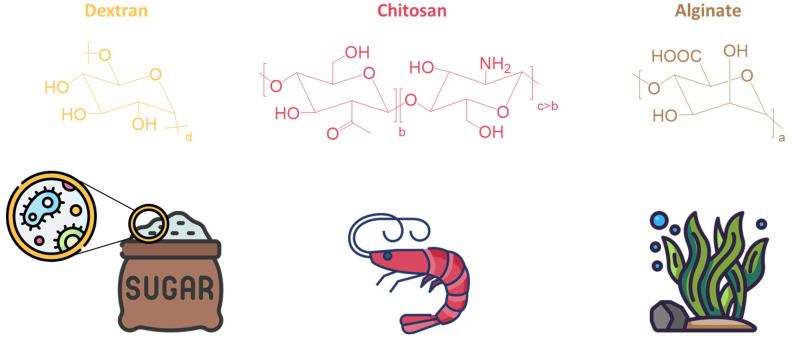
Chemical formulas of dextran, chitosan, and alginate.

Surgical glues can also be prepared from polysaccharides such as dextran, chitosan, or alginate (Figure 17) [97,98,99]. Since polysaccharides are composed of natural sugar building blocks, they are generally biocompatible and do not cause inflammation. Although some polysaccharides are naturally inert and non-degradable in the body, they can become hydrolytically unstable through chemical modifications. Additionally, some polysaccharides, such as chitosan, have intrinsic antibacterial properties that reduce microbial infections after surgery. This section will discuss examples of polysaccharide-based tissue adhesives.

(a) Dextran

**Scheme 8:**
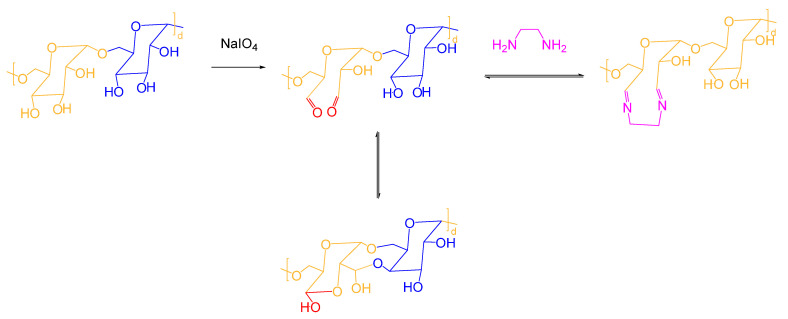
Oxidation of dextran into dextran-aldehyde, followed by its reaction with the amines of the body.

Dextran is a polymer made of glucose units linked by α-1,6 bonds and which is obtained from the enzymatic action of bacteria (lactobacillus, streptococcus…) on saccharose. This biocompatible polymer is water-soluble and physiodegradable, it can be excreted from the human body by the liver, kidneys, spleen, and colon after its degradation by α-1,6-glucosidase. Due to a high amount of hydroxyl groups in its backbone, dextran has a high water-binding capacity. It is thus utilized as hemostatic sponges. Indeed, lyophilized oxidized dextran has a large pore size (30–50 μm) allowing it to absorb blood and then control bleeding. Thanks to its rich concentration of free hydroxyl groups, dextran can also be post-functionalized and cross-linked. In the case of adhesion, the hydroxyl groups of dextran are oxidized into aldehydes. These later can further react with amine-containing crosslinkers (gelatin, chitosan…) and/or the amines in the body to create bio-adhesion (Scheme 8). Dextran generates good adhesion, better than fibrin, due to the covalent nature of the imine bond, even in moist environments. On the other hand, the presence of aldehyde can be a disadvantage [2]. Dextran undergoes a fast physiodegradation (∼3 days) by hydrolysis of the imine bonds and polysaccharides. Its degradation products are also biocompatible. The main drawback of such a system is the necessity to use a large quantity of sealant to close the incision, because of its too rapid degradation for a lower amount (less than 24 h). This can lead to foreign body reactions [24]. Actamax™, which is based on oxidized dextran and PEGylated amines, received conditional approval from the FDA. Its adhesion strength can reach 100 kPa.

Dextran is frequently used to crosslink chitosan or gelatin. For that reason, there is no research based on dextran chemistry for surgical adhesives.

(b) Chitosan

Chitosan is a polyoside composed of randomly distributed D-glucosamine and N-acetyl-D-glucosamine. It is produced commercially by the deacetylation of chitin, found in the exoskeleton of arthropods (insects and crustaceans) or the cell wall of fungi. Chitin is an insoluble linear polysaccharide, chemically similar to cellulose even though one hydroxyl group on each monomer is replaced by an acetylamino group. In medicine, chitosan is used as a hemostat, enhancing the coagulation process. Since red blood cells are charged negatively, chitosan which is charged positively, attracts them, in contact with blood, the pad becomes extremely adherent, and a seal over is generated. Chitosan is non-toxic, biocompatible, and physiodegradable by the action of enzymes in a biological environment, and it is not greatly soluble in water. Additionally, it also possesses antimicrobial and bacteriostatic properties (algae, bacteria, yeast, and fungi). Thanks to the amino groups electrostatic attractions are generated with the collagen of the tissue. For these reasons, chitosan is used for wound healing and tissue adhesive (hydrogel, sponge or fibers). Chitosan-based wound dressings such as Chitoflex pro^®^ or bandage pro^®^ (HemCon) are used as hemostats for extreme external trauma. An outer wrap bandage must be applied on the pad and can be removed after 48 h with saline or water. Dental dressing^®^ are pads that can be used in oral treatments (gingival grafts and tooth extractions). As tissue adhesive, chitosan is mixed with aldehydes or isocyanates, which react with the amine of the chitosan.

**Figure 18:**
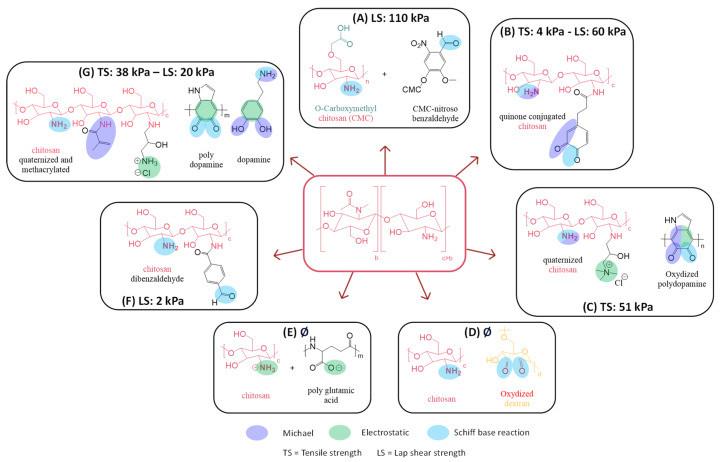
Reagents and types of chemistries utilized by researchers to synthesize chitosan-based surgical glues (n, b, c: nb of repeating units).

In the recent research, chitosan glue has been based on different kinds of chemistries, represented in Figure 18, including Michael addition [142,143,144], Schiff base reaction [142,143,144,145,146,147] and electrostatic interaction [15,143,144]. In this regard chitosan was functionalized [142,145,147], quaternized [143,144], or acrylated [143,144] In some examples, these chemistries are utilized in synergy. All those formulations exhibit biocompatibility and adhesion on tissues ranging from a few kPa (B [143], F [147]) up to tens of kPa (A [142], C [145] and G [144]). Jayakrishnan et al. (D [146]) did not test adhesion and Porta et al., (E [15]) tested the adhesion on wood and aluminum and obtained lap shear strength adhesion of only 3 Pa. It is worth noting that for a same kind of chemistry (e.g., Schiff base reaction), the adhesion values can be significantly different such as in A (110 kPa) and F (2 kPa). Even though they are low, those values are promising since they surpass fibrin glue’s adhesion for some of them.

(c) Alginate

Alginic acid is a naturally occurring polysaccharide found in brown algae. It is a copolymer of mannuronic acid (some units are acetylated) (M units) and guluronic acid (G units) linked by β1-4 bonds. It is hydrophilic and forms a viscous gum when hydrated [4]. With sodium or calcium, its salts are known as alginates. Alginates are biocompatible, edible, and low cost. They are used in the food, pharmaceutical, and cosmetics industries as thickening and gelling agents, known by the code E400-405. Alginates have also been used as wound dressing in the presence of calcium ions, since it forms a crosslinked polymer gel. Calcium ions of the wound dressings and sodium ions from the blood serum exchange. When sufficient sodium is reached on the dressing, the fibers swell and form a gel. The gel created is highly hydrophilic, which limits wound secretions and minimizes bacterial contamination. Its hydrogel is also used for bone reconstruction and tissue regeneration. Commercial wound dressings based on alginate are commercialized such as Tegaderm Alginate™ (3M) or Coalgan^®^ (Laboratory Brothier, Nanterre, France). These devices are capable of absorbing blood up to 20 times their weight. It is available as gauze, wick, and powder. It can be used as wound dressings or to stop nosebleeds. Additionally, it is not reserved for professional use and can be found in pharmacies.

**Scheme 9:**
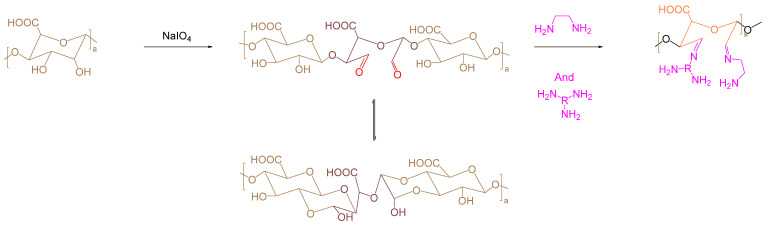
Oxidation of alginate backbone utilizing sodium periodate, potential degradation reaction, and reaction leading to adhesion with the skin and cohesion of the network with the amine crosslinker (a: nb of repeating units).

Since it has low toxicity and similarities with the extracellular matrices of living tissues, alginate-based adhesives have been intensively studied [148,149,150,151,152,153,154,155,156,157]. Due to a lack of lyases (bond-cleaving enzymes), alginate is inert to the body, meaning that it is non-biodegradable. For this reason alginate is often oxidized for application in the body. This leads to the cleavage of the C-C bond of the cis diol generating hydrolytically sensitive bonds. As seen in Scheme 9, this oxidation leads to the formation of aldehyde functions. Therefore, the crosslinkers utilized are amine-based. This formulation provides better adhesion than fibrin glue [6].

**Scheme 10:**
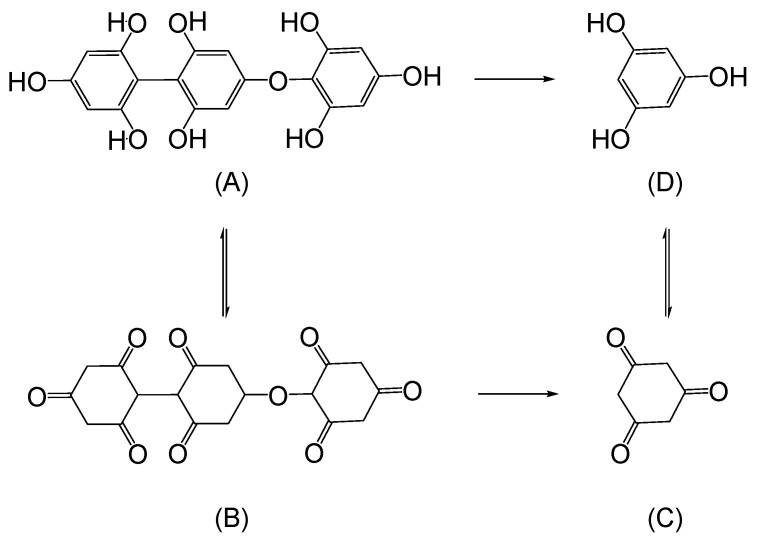
Chemical structure of brown algae polyphenol (**A**), oxidized polyphenol (**B**), phloroglucinol (**D**), and oxidized phloroglucinol (**C**) [160].

Brown algae also secrete polyphenols that can be crosslinked after an enzymatic catalysis [95], Scheme 10A [158]. In 2008, Bianco-Peled et Bitton [159,160] published their results on algae polyphenols-alginate-calcium adhesives. They studied four formulations (Scheme 10), the first one with polyphenol, the second with the oxidized polyphenol, and the third and fourth with native and oxidized phloroglucinol. The oxidized molecules were thus able to react with the amines of the body via Schiff base reaction. These adhesives exhibited good adhesive strengths and showed better adhesion toward hydrophobic surfaces. In order to be used as surgical glue, the adhesion on hydrophilic surfaces must be improved. They obtained better adhesion with the non-oxidized formulations. Among the phenols, the monomeric one provided a higher strength on several substrates, including porcine tissue (17–25 kPa). This formulation received the CE mark in 2017 and is now used as a surgical sealant to reinforce anastomoses during surgery, in order to prevent fluid or air leakage (SEAL-G^®^—Sealantis, Sealantis, Malaysia). In colorectal surgery, it is 91.4% leakproof. This formulation can be stored at room temperature, and its gelling time is about 60 s. This sealant is durable and flexible, and is absorbed over time (50% degradation after 3 months).

**Scheme 11:**
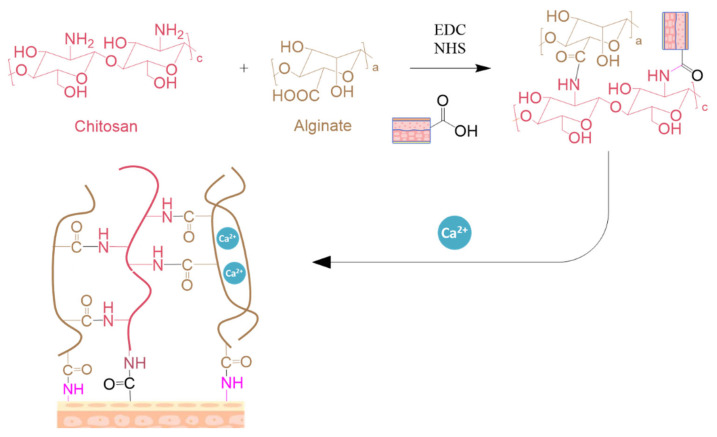
Reaction of chitosan on biological amine and on alginate thanks to EDC/NHS (a, c: nb of repeating units).

In order to improve the adhesion of chitosan glues, researchers are utilizing it with alginate. Indeed, its carboxylic acids can react with the amines of chitosan in the presence of EDC/NHS (Scheme 11). Moreover, when calcium ions are added, an electronic network is thus generated [161,162].

##### C. Conclusion

To conclude this section about biological and biochemical adhesives, it is noteworthy to mention that all of them have their own specific advantages, which can be a plus according to the targeted application. Protein based surgical glues such as fibrin are widely utilized in surgery, mostly to enhance the natural healing process, mimicking the natural coagulation. Even though they are trustworthy, their adhesion strength remains low, limiting their utilization to temporary adhesion or hemostasis. On the other side, surgical glues based on gelatin exhibit good biocompatibility and physio-degradability, but their strength is also low. On the other hand, since they possess a stronger adhesion, the research about albumin based surgical tissue adhesives is very active in order to continue to increase their adhesion and enhancing their safety. On the side of polysaccharide based surgical adhesives, chitosan and alginate have good potential thanks to their ability to be crosslinked and functionalized. Research on dextran is still ongoing for other applications. Even if these polysaccharides possess good biocompatibility and physiodegradability, their strength remains low, and major work has to be completed in order to increase this feature, to be able to compete with protein-based formulations. Concerning the commercial applications, fibrin glues are well established on the market and widely utilized (e.g., Tisseel). In conclusion, fibrin seems to be the best solution available while polysaccharides are still under research, considering their high potential. 

#### 5.2.2. Biomimetic

**Figure 19:**
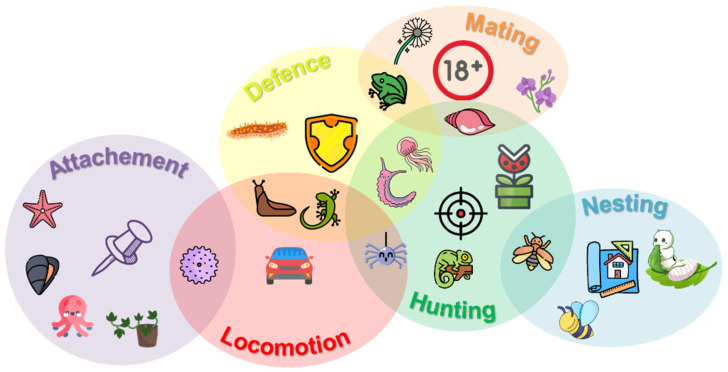
Classification of animals, plants, and insects with adhesion abilities according to their purposes.

For millions of years, evolution has led to the development of diverse kinds of adhesives and slimy substances in both animal reign and plants. These bio-compounds have evolved to help organisms survive, adapt, and reproduce in various environments. Often, these substances serve multiple functions simultaneously, providing a range of benefits. Scientists have therefore taken an interest in these substances and have begun studying them to understand and replicate them, in order to develop biocompatible adhesives. Consequently, surgical glues are inspired by molecules produced by living organisms (mussels, slugs, etc.) or from physical particularities owned by some species (geckos, octopuses). Some of them are depicted in Figure 19 and their mechanisms of adhesion as well as their purposes are summarized in Table 3. For example, mussels and clams secrete an adhesive protein that helps them to settle on rocks for instance. Geckos possess adhesive toepads allowing them to walk on smooth or inverted surfaces. Slugs also leave adhesive proteins on their way. Inspired by the unique properties of these naturally occurring molecules, researchers now tend to develop tissue adhesives that mimic the natural adhesion of these organisms [163,164].

##### A. Mussel/Clams

**Scheme 12:**
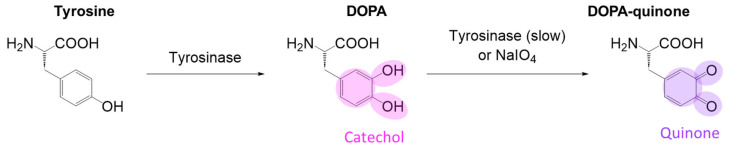
Hydroxylation of tyrosine to DOPA then oxidation to DOPA-quinone.

Mussels are currently intensively studied for their ease of sticking in wet and wavy environments [165,166,167,168,169,170,171,172,173]. In this regard, the mussel-inspired hydrogels account for a major class of bioadhesives, wherein catechol groups play a dominant role in establishing adhesion. Their adhesion relies on the creation of a byssus (bundle of filaments produced by mussels that is attached to a solid surface). The byssus contains specific proteins that have incredible adhesion. Scientists identified six mussel proteins. One of these proteins called adhesin, is a component also found at the surface of germs, allowing for the bacteria to adhere to their host while infecting them. Adhesin bonds strongly to surfaces by forming chemical and hydrophobic bonds. Although these proteins exhibit various chemical properties, a particularly significant component is redundant: the catecholic amino acid, 3,4-dihydroxyphenylalanine (DOPA). Indeed, mussel proteins also contain tyrosine which in presence of tyrosinase produces DOPA. The oxidation of this later leads to a quinone amino acid (Scheme 12).

**Scheme 13:**
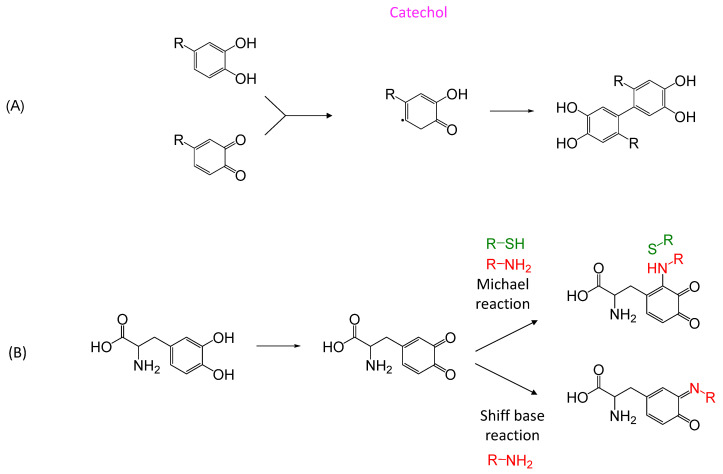
(**A**) Dimerization of the catechol/quinone and (**B**) Michael or Schiff base reactions with amines and thiols of the body.

Catechol and quinone are known to have adhesive properties [144,145]. This is why, thanks to these special protein-based adhesives, mussels can adhere better than synthetic or other natural glues. Indeed, mussels can attach to surfaces such as Teflon^®^. Moreover, this adhesive is water-insoluble, biocompatible, and does not generate an immune response [24]. Because the extraction of the adhesive proteins is uneconomical and not scalable (around 10,000 mussels are required to get 1 g of the foot adhesive protein) [41], this adhesive is not commercialized yet. In this context, scientists are now working on the synthesis of a glue that mimics these proteins, to make surgical water-resistant adhesives. The main bottleneck is that the synthesis of byssus is complex and requires specific conditions. In this regard, adhesives containing L-DOPA [166,169,172], dopamine [174,175,176], dihydroxy-caffeic acid [177], etc., have been synthesized. In these types of adhesives, adhesion arises from weak molecular interactions (such as hydrogen bonding), while the network cohesion relies on covalent bonds that are obtained either by direct crosslinking of the quinone with itself via dismutation or tautomerization (Scheme 13A), or by Michael reactions with nucleophiles (amines and thiols of the body) or Schiff base reactions (with amines) (Scheme 13B) [4].

**Figure 20:**
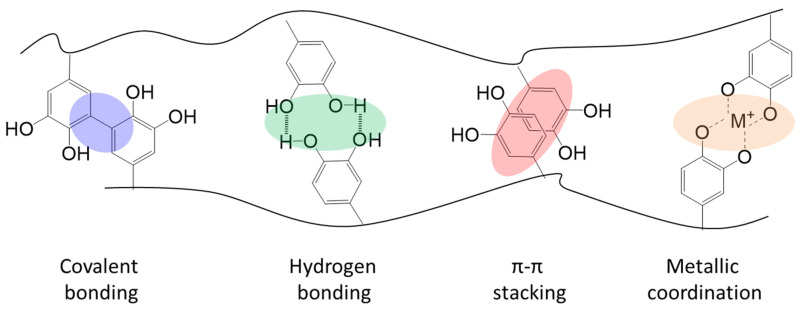
Possible interactions of catechol with itself.

Aside from crosslinking, the network cohesion is reinforced because catechol can undergo hydrogen bonding, π-π stacking, and metallic coordination, as presented in Figure 20.

These types of formulations exhibit adhesion up to eight times that displayed by fibrin. The curing time can be tuned to be in a range from a few seconds to several minutes. These adhesives are completely degraded after a month in physiological conditions. On the other hand, mussel-inspired adhesives can irritate the skin [102]. Moreover, the action of tyrosinase in the oxidation of DOPA to quinone is very slow, requiring the addition of chemical oxidants (e.g., sodium periodate) to accelerate the oxidation rate. In the body, the presence of enzymes, varying pH, natural redox agents, etc., can also modulate the reaction. The biocompatibility of these compounds is unclear and raises concerns about toxicity. In addition, the storage of catechol requires low pH and anaerobic conditions. The potential autooxidation, which leads to the generation of oxygen species (such as H_2_O_2_), must also be considered, as it can potentially damage tissues. For all these reasons, catechol-based surgical glues have not been commercialized yet.

##### B. Gecko

Although mystifying scientists for decades, the mechanism of gecko adhesion to walk on vertical or inverted surfaces, whether rough or smooth, has been now unveiled. The adhesion of geckos relies on the hundreds of thousands of micro-tips on their feet. Indeed, gecko feet are covered with fibrillar arrays (setae) that maximize the interfacial adhesion; each seta possesses numerous spatulas. This allows for the multiplication of Van der Waals and capillary forces [102], which together control the adhesion of these spatulas to surfaces.

Adhesives that imitate the geckos’ feet have become a major focus of research. The most widespread method for creating these synthetic mimetics involves designing surfaces with densely packed nanoscale pillars to achieve interactions with the targeted surface, similar to those of geckos. Gecko-inspired dry adhesives are then synthesized.

Indeed, they allow the repositioning of the material without leaving residues on the substrate. This kind of adhesive can be used to replace PSA, thus eliminating primary irritation, painful detachment, and supplying reusability. For now, this type of patch is not adherent enough (1.3 N.cm^−2^) [58].

**Scheme 14:**
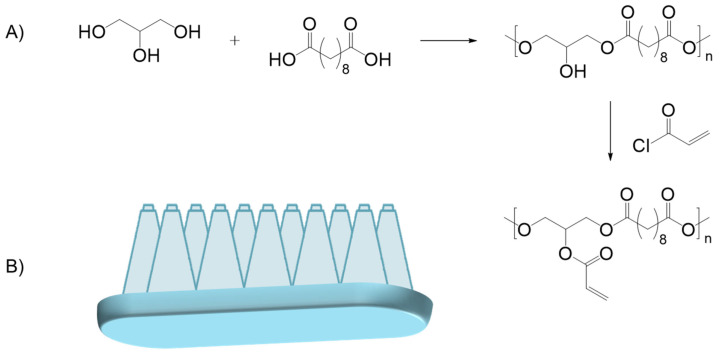
Synthesis of poly(glycerol-sebacate acrylate) from glycerol, sebacic acid and acryloyl chloride (**A**) and representation of the nanopatterned PGSA elastomers (**B**).

However, this adhesion based on the multiplication of Van der Waals forces is lost when exposed to wet conditions. In this regard, tissue-reactive molecules have been synthesized. In 2007, Ferreira et al. [178], published findings on photocurable elastomers from poly(glycerol-co-sebacate acrylate) (PGSA) (Scheme 14A). This polymer is biocompatible and biodegradable and its degradation products are non-toxic. In 2008, they published research on gecko-inspired polymeric nano-patterns [179]. In order to mimic the nano-topography of the gecko feet, they cast PGSA on nano-mold cavities (Scheme 14B). To ensure that the polymer adheres in a humid environment, they applied a layer of oxidized dextran, containing aldehyde functions, to their model pattern, to get adhesion with the body thanks to Schiff base reaction. As seen previously, the aldehydes are able to react with the amines of the body to lead to imines. In addition, to lead to matrix cohesion, the aldehydes can react with the free hydroxyl groups of the glycerol, resulting in hemiacetals. The adhesion was tested in vitro on the porcine intestine and in vivo on the rat abdomen. They also tested the adhesivity of the “flat-unpatterned” formulation, which was 2-fold lower than the one of the casted polymers. In 2014, they compared it with cyanoacrylate (CA) in a dry and blood-exposed environments [180]. In a dry environment, their formulations achieved 0.5, 1.8, and 2 N/cm^2^ after 1, 5, and 30 s, while CA reached almost 4 N/cm^2^. Conversely, when exposed to blood flow before curing, CA exhibited almost no adhesion while the PGSA formulation showed an adhesion of 1.2 N/cm^2^ after 5 s. A French start-up (Gecko Biomédical/Tissium, Paris, France) obtained the CE mark in 2017 for their Setalum™ Sealant. Their formulation relies on a high viscosity PGSA prepolymer (10/15 Pa·s), curable with visible blue light and that is used to reinforce bowel sutures. The acrylates create a 3D network which bring cohesion, the adhesion relies on weak interactions such as Van der Waals. Therefore, this adhesion is reversible, similar to that in gecko feet. The resin can also be used to build 3D devices (e.g., microfibers). This polymer degrades in 9–12 months. In 2019, it was preclinically and clinically tested. The glue was found to have good biocompatibility and bioresorption in animal studies. Furthermore, the first trial on humans demonstrated that the sealant was harmless and an effective alternative to controlling hemostasis in vascular surgery [181].

##### C. Slug/Snail

The mucus secreted by slugs, when threatened by a predator, is composed of glycosylated proteins (positively charged), heparan-sulfate-like mucopolysaccharides (such as hyaluronic acid—negatively charged), glycolipids, and other biomolecules [182]. It is 95% water-based. This mucus prevents the slug from being removed from the surface by the predator. Although not yet clearly understood, the mechanism is probably based on a double network, where the positively charged molecules provide a largely crosslinked network, and the negatively charged species form an extensible network with positive ions such as Ca^2+^ or Mg^2+^. Additionally, this viscous gel can adhere to surfaces thanks to molecular interactions such as Van der Waals forces, electrostatic interactions, and hydrogen bonding. This mucus is capable of resisting humidity and friction [183]. For these reasons, some research groups have begun studying this gel and developed adhesives based on slug [182,184,185,186].

##### D. Other Naturally Occurring Adhesives

Eggs are naturally abundant products that are nontoxic, biocompatible, biodegradable, and low cost. The white part of the egg is able to exhibit adhesion [1]. Xing et al. [187], extracted the albumin from the egg white, allowed it to dry overnight, and ground it into fine powder. Afterward they mixed it with twice deionized water and coated this mixture on PCL nanofibrous membrane. They obtained an adhesion of 56.2 ± 15.2 kPa on pigskin, comparable to that of cyanoacrylate (55.4 ± 19.6 kPa), and higher than that of fibrin (24.0 ± 9.3 kPa).

Spiders are able to secrete a sticky substance to catch their prey. This glue maintains its adhesiveness under wet conditions. It is composed of glycoproteins containing charged amino acids that can remove the interfacial water of the prey and then to form adhesion [6]. This mechanism was used to synthesize a dry double-sided tape based on biopolymer (gelatin or chitosan) and crosslinked poly(acrylic acid)—PAA, grafted with NHS-esters [188]. The PAA can absorb water and form hydrogen bonds. The NHS, in turn, allowed crosslinking with the amines of the body. This tape allowed to reach a tensile strength of ≈125 kPa on porcine skin, while all the commercially available tissue adhesives tested was not able to reach 50 kPa (bioglue ≈ 45 kPa, dermabond ≈ 35 kPa coseal and duraseal ≈ 10 kPa, tiseel ≈ 5 kPa). The adhesion was also tested on small intestine and muscle (≈80 kPa), stomach and heart (≈75 kPa), and liver (≈20 Kpa). The biocompatility tested on mouse fibroblasts yielded results comparable to those of the control medium.

Some plants are also able to undergo adhesion, English ivy is able to adhere to vertical surfaces (walls, for instance) thanks to a yellow sticky adhesive. This adhesion is so strong that in some cases, the wall collapses before the adhesion is broken. This adhesive is based on spherical nanoparticles of arabinogalactan proteins. These nanoparticles spread across the interface and permeate the surface, maximizing close contact and improving mechanical interlock. After the water present in the adhesive evaporates, the nanoparticles become more concentrated, leading to a strong bond. Additionally, calcium-driven electrostatic interactions between the carboxyl groups of arabinogalactan proteins and pectic polysaccharides in the aqueous phase help harden the adhesive, enhancing its mechanical properties [6]. Zhang et al. [189], were able to obtain lap shear adhesion up to 550 kPa on glass slides with such a formulation. Liu and his team [190] obtained up to 85 kPa on porcine skin, while Tisseel gave around 5 kPa. Polyphenol groups (tannins) from plants can also be used [191].

Sandcastle worms secrete a sticky substance composed of oppositely charged proteins. These are later complexed by magnesium and calcium ions. The positively charged proteins contain basic traces with an amine side chain, while the negatively charged proteins include acidic phosphoserine residues. The worms package their glue into microgranules and then release them into the seawater. In the presence of electrolytes and high pH, the granule membrane breaks, and the glue bonds to sand particles [192]. They obtained lap shear strength on glass between 700 and 1000 kPa on glass and from 500 to 2500 kPa on PMMA substrate. On cow skin they achieved ≈ 45 kPa, while fibrin gave ≈ 20 kPa and PEG-based glue ≈ 25 kPa.

##### E. Conclusions

In this section, several biomimetic solutions were explored, in particular DOPA-based surgical adhesive, mimicking mussel proteins, nanopatterns mimicking the gecko feet, and other substances produced by organisms such as spiders, slugs, ivy, and sandcastle worms. However, in terms of viability, the most promising are the solutions based on DOPA and on nanopatterns. DOPA-based adhesives show great adhesivity in wet environments, which is a required feature for surgical glues. Nanopatterns on their hand, allows a high mechanical adhesivity without damaging tissues. Other solutions, although innovative, remain limited in terms of scaling. Adhesives inspired by spiders, ivy, or sandcastle worms require some optimization to be able to compete with the properties of DOPA-based adhesives.

#### 5.2.3. Synthetic Surgical Glues

Even though natural-based adhesives present some advantages in terms of biocompatibility and low toxicity, they suffer from limitations such as low adhesivity in wet environments [2], variability of the properties depending on the batch, fast degradation, etc. This leads research to focus on synthetic adhesives such as polyurethane, cyanoacrylate, and PEG, for instance. Synthetic adhesives answer to major stakes, such as biocompatibility, the reduction of the allergy risk, better performance, and adaptability. Indeed, these adhesives are versatile. Thus, the gelling time, the flexibility, and the degradation rate can be tailored.

##### A. Polyurethanes—Polyurea

**Scheme 15:**
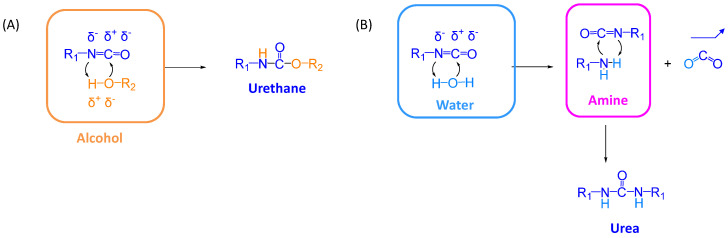
Reactions of isocyanate with alcohol (**A**) or water and amine (**B**).

As mentioned above, polyurethanes are based on the polymerization of diols or polyols and di- or poly-isocyanates (-N=C=O). Due to the electronegativity difference between these atoms, isocyanates are very reactive with any nucleophile. Thus, the negatively charged nitrogen bonds with the hydrogen from the alcohol while its oxygen links with the positively charged carbon, forming a urethane bond (Scheme 15A). Isocyanates can also react with water (generating carbon dioxide and an amine) and amines. In this case, the resultant amine can further react with another available isocyanate, forming a urea bond as the final product (Scheme 15B).

Polyurethanes (PU) are a large family of polymers used in a wide range of applications, including foams, coatings, elastomers, and adhesives. Most commercial PUs are synthetically produced from petroleum-based feedstocks, such as methylene diphenyl diisocyanate (MDI) or toluene diisocyanate (TDI), and polyether or polyester polyols. These materials are generally not biodegradable and may generate degradation byproducts of questionable toxicity, particularly in environmental contexts. However, in the biomedical field, specific PU formulations have been designed to be biocompatible, biodegradable, and non-toxic, using alternative diisocyanates such as HDI or LDI and polyols such as PEG or polyester diols derived from renewable sources. These tailored PUs exhibit completely different properties and degradation behavior compared to conventional industrial Pus.

**Scheme 16:**
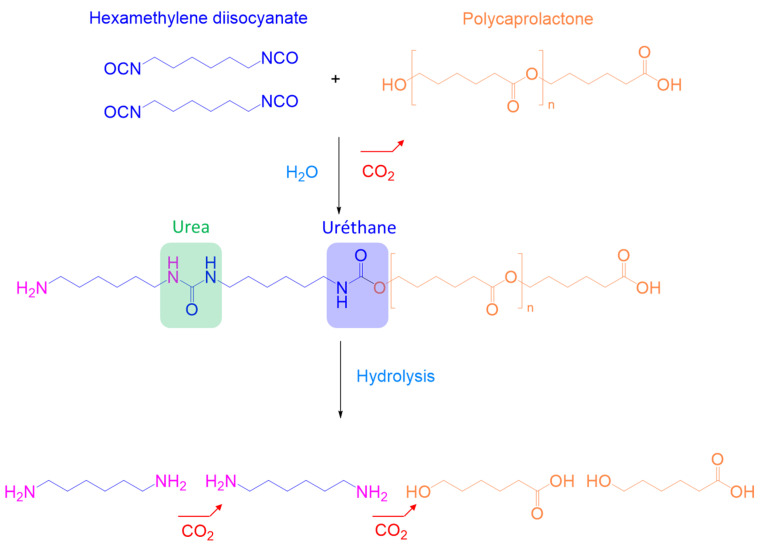
Polyaddition of diisocyanates (hexamethylene diisocyanate) on polyester-diol (polycaprolactone) in the presence of water leading to urethane, urea, and generation of CO_2_, then hydrolysis of the urethane, urea, and ester groups with generation of CO_2_ (n: nb of repeating units).

PU is used in various fields including biomedical applications (e.g., catheters, short-term implants) [193]. Indeed, PU is known to be biocompatible. As a matter of fact, polyols can be polyether, polyester, or vegetable oil-based, and these molecules are inert to the body, non-toxic, and do not generate an immune response. In addition, they can be degraded by hydrolysis and subsequently metabolized. Moreover, their degradation products are not toxic either. To illustrate this point, Scheme 16 represents the reaction of two molecules of hexamethylene diisocyanate (HDI) with polycaprolactone (PCL) in the presence of water and their hydrolysis products [194,195].

**Figure 21:**
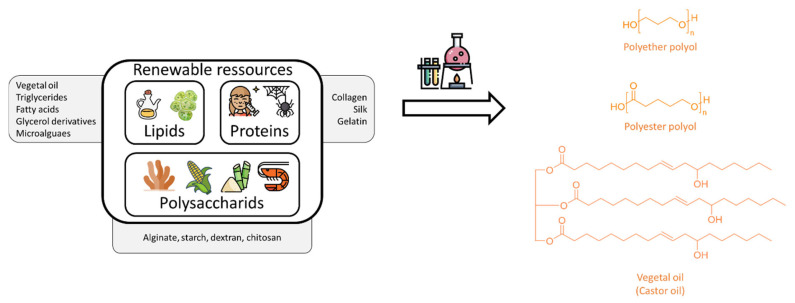
Renewable sources of polyols.

Thanks to their adhesive features toward numerous substrates [91], polyurethane was extended to the field of adhesives in 1940. Thus, they started to be considered promising as surgical glue as well. In this regard, PU biocompatible sealants were first reported in 2003 [193], and are now used as wound closure adhesives and surgical glue. For instance, Adhesys Medical synthesized a topical adhesive (MarCutis) based on polyurethane. MarCutis remains on the skin for up to 10/12 days, then falls off. It received the CE mark in 2018. In addition, to their adhesivity, biocompatibility, and physio-degradability, PUs possess good mechanical properties, are inexpensive to produce, and are increasingly biobased (cf., Figure 21) [196].

**Scheme 17:**
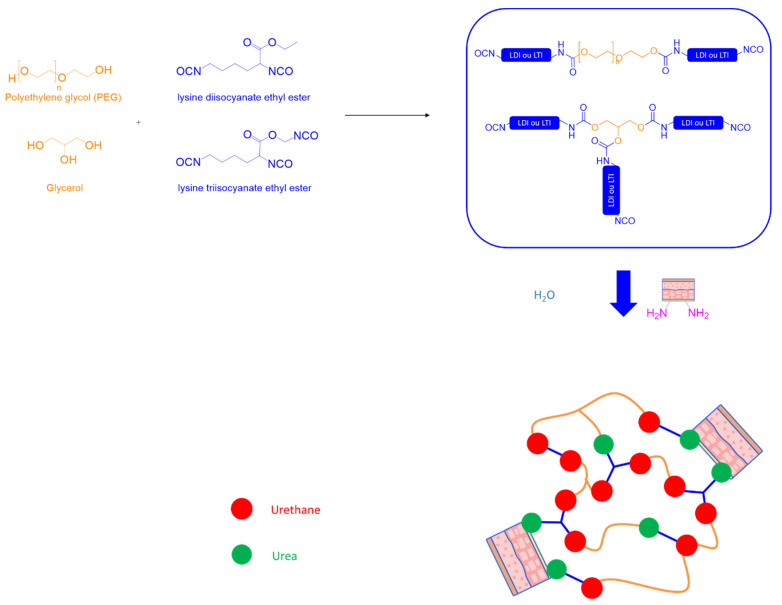
Synthesis of isocyanate-terminated prepolymers, then crosslinking with water and amines.

To date only one PU-based technology is commercially available as surgical glue; Tissuglu^®^ (Cohera, Vienne, France, patented and CE Marked in 2011 and approved by the FDA in 2015) [197]. This was developed to avoid seroma in abdominal surgery by gluing the underlying layers of the abdominal wall to the abdominal skin tissues, limiting the dead space. The research of Beckman et al. led to several patents [105,198,199,200,201,202]. Its first purpose was to replace drains in abdominoplasty. This one-component adhesive is based on a polyethylene glycol (PEG)/glycerol prepolymer terminated by LDI/LTI (lysine di and triisocyanate)—Scheme 17. When in contact with humidity, amines are generated, these later and the amines of the body further react with the remaining isocyanates, forming a poly(urethane-urea) network that is covalently bonded with the tissues. The gelling time of such a formulation is 25 min. This time is too long to meet the requirements of surgery. All the degradation products generated by hydrolysis are non-toxic (e.g., lysine, PEG, ethanol, etc.). On the other hand, this adhesive also postpones the healing and can generate redness and hematoma. PU is also used as a sealant and hemostat. For example, they are used in order to avoid leakage of blood in the body in vascular graft. For instance, Cohera used the same technology of Tissuglu^®^ in addition to triethoxysilane in order to develop another surgical glue that was meant to be used in conjunction with standard closure methods in gastrointestinal surgery (Sylys). During the clinical study, no leakage was reported. In 2015, this adhesive received CE mark, and it has started to be evaluated in the USA.

Concerning the research, it is mostly centered on polyurethane/polyurea. Indeed, polyol prepolymers are functionalized into isocyanate and then crosslinked with the water from air humidity or the amines of the body. For example, Xia et al. [203] synthesized castor oil/PEG-based PU with biocompatible and biodegradable properties. The isophorone diisocyanate (IPDI) extremities allowed it to cure at room temperature, thanks to the ambient humidity, within 7–25 min. They evidenced that the curing time was shortened by increasing the castor oil amount. This glue provided a lap shear strength of 43 kPa. Other researchers used hexamethylene diisocyanate (HDI) or dimeryl-diisocyanate (DDI) and obtained similar strengths [204,205,206,207]. These glues provide better adhesion than fibrin (2–18 kPa) but lower than cyanoacrylate (650 kPa). On the other hand, the presence of urea can generate rigidity and a lack of control over the polymerization rate (adding an amine to an isocyanate is instantaneous). For this reason, the synthesis of a surgical glue based only on urethane can be relevant.

For this reason, several research groups are working on 2-component surgical glues. The first component is isocyanate/isocyanate-terminated prepolymers, and the second is polyols. For example, Wendels et al. [13,14,205] synthesized different generations of two-component surgical glues utilizing cotton oil or poly(hydroxy-alkanoate)—PHA, functionalized either with HDI, DDI, LDI, pentamethylene diisocyanate (PDI), or diphenylmethane diisocyanate (DMDI). Then they placed them in a hot press at 80 °C for several hours with short diols such as butane diol to achieve full crosslinking of the formulation. The obtained adhesion was comparable to fibrin (2–18 kPa), which was lower than that of polyurethane-polyurea. They also demonstrated that the shorter the diol, the higher the adhesion. Unfortunately, gelling times for such formulations have not been studied.

As mentioned earlier, the major stakes for a surgical glue are its ability to cure at body temperature in a short period. The most important drawback that comes with a polyurethane formulation is the lack of reactivity of isocyanate towards alcohol at room temperature. Since heating of the application site is impossible and since the gelling time has to be achieved within ten minutes, catalysts are then required [208,209]. In this context, researches now focus on catalytic systems, such as tertiary amine, as reported by Zheng [210]. Using this type of catalyst, they were able to get 5 min of gelling time on castor oil derivative/DMDI and poly(methylene poly-phenyl isocyanate)—PAPI formulations. These polymers exhibited 0.8–2.2 MPa lap shear strength, which is higher than cyanoacrylate. Another way to benefit from polyurethane advantages such as biocompatibility and biodegradability, without the curing time drawback, is by using urethane prepolymers ended with acrylate moieties. Some are using urethane dimethacrylate (UDMA) [211], while other are synthesizing urethane prepolymers, which are terminated by alcohol moieties instead of isocyanate, and reacting them further with isocyanate ethyl-methacrylate (IEMA) [212,213,214]. Ates and his team used cyclodextrin, PEG, and HDI and were able to obtain adhesive after 5 min of irradiation. These adhesives possess better adhesion than the classical PU, ranging from 1.3 to 4.3 MPa. Another way to shorten the curing time of urethane when using isocyanate prepolymers is to make them react with thiol, thanks to click chemistry. Without a catalyst, the reaction proceeds in 3 min with a formulation made with PEG, star polyesters, and HDI, and allows us to obtain lap shear adhesion of 110 kPa [215].

**Scheme 18:**
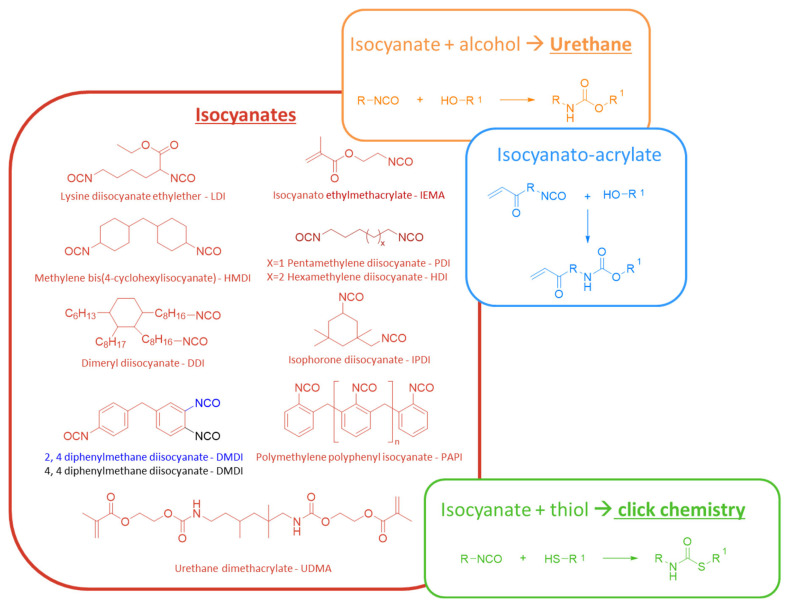
Formulas of the isocyanates and the different kinds of chemistries described in this section (n, x: nb of repeating units).

To conclude, there are different ways to obtain polyurethane surgical adhesives without the disadvantage of their long curing time at room temperature (Scheme 18). The main advantage of using PU is the possibility to tailor the adhesive with the desired features. For instance, they are all biocompatible and offer adjustable in vivo degradation time. They all have their drawbacks, which is why only one formulation is commercially available, even though several patents exist [216,217,218]. However, day after day, the research comes closer to the perfect PU surgical glue.

##### B. Cyanoacrylates

**Scheme 19:**
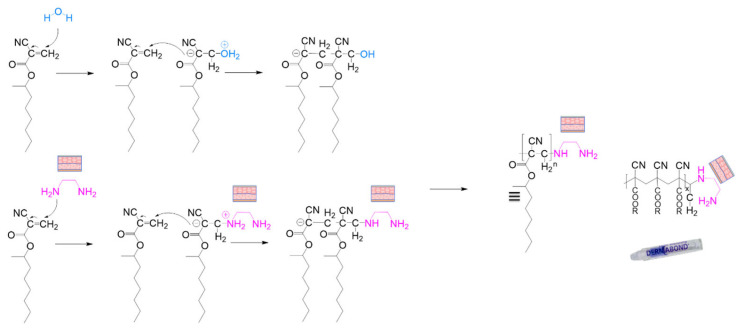
Polymerization of 2-octyl-cyanoacrylate in the presence of water and amines of the body.

Discovered and patented in 1942 by a team of researchers of Goodrich [219], cyanoacrylates were commercialized in 1951 by Eastman as Eastman #910-adhesive [220]. They are the most widely used adhesives in both everyday life and medical applications due to their unique chemical and physical properties. These adhesives cure rapidly without the need for a catalyst, offering strong adhesion across a wide variety of substrates. Remarkably, they can bond to target surfaces within 5–6 s upon contact with basic substances such as water, blood, body tissues, or moisture, making them highly effective in diverse conditions. Cyanoacrylates were first used at an industrial scale, then became available in DIY stores (they are the family of the ‘super glue’ we can find at home) [220]. Developed by a German chemist in the early 50’s, cyanoacrylate wound closure adhesive was clinically utilized by a British surgeon in 1959 [221]. The only difference between DIY and surgical glues is that ethyl-cyanoacrylate is found in the super glue, and 2-octyl (Dermabond^®^—Ethicon, Raritan, NJ, USA, and Liquiband^®^—Medtronic) or n-butyl-2-cyanoacrylate (swiftset™—Covidien, Dublin, Ireland, and Leukosan^®^ adhesive—Leukoplast, BSN Medical, Charlotte, NC, USA) are used for surgery because they are less toxic (by increasing the length of the side chain, the volatility and toxicity decrease) [222]. Increasing the length also slows down the polymerization rate, reduces the strength of the adhesive, but increases the flexibility, so the breaking strength. Cyanoacrylates have been accepted since the 80s in Canada and Europe, and 2-octyl-cyanoacrylate was approved by the US FDA in 1998. Firstly classified as a class III device, it is now classified as class II [223,224]. Cyanoacrylates have sealant and hemostatic properties as well as antimicrobial activity [22]. For these reasons, cyanoacrylates can be used as wound closure adhesives. As seen in Scheme 19, the monomers polymerize quickly (gelling time between 5 and 40 s, total polymerization around 60 s) and at room temperature, thanks to a small amount of water present at the surface of the skin or even simply with the moisture contained in the air. This quick mechanism is due to the nitrile group linked to the double bond, which is an electron-withdrawing group, allowing the anionic polymerization even by weak bases such as water or amines [59,220]. During its polymerization, the amine or thiol of the living tissues can be added thanks to a Michael addition (aza- or thia-Michael); this plays a key role in the adhesion mechanism. The adhesion of acrylate also relies on interlocking. Indeed, the first mechanism involved in acrylate bonding is its penetration into the tissue surfaces (cracks and channels) and its crosslinking with them. On the other hand, the reaction is quite exothermic (>80 °C), and the operative area has to be kept dry [220]. Moreover, when reacting with the tissues, cyanoacrylate monomers are vaporized into a sensitizer and irritative smoke. Thus, good aeration is needed while utilizing this glue. Additionally, to this downside, even if the rapidity of this polymerization can be a plus, it can also be a drawback, if the surgeon messes up, it cannot reposition the edges of the skin. It also has to be taken into consideration that the viscosity of this kind of glue is very low (10^−3^–10^−2^ Pa·s). Cyanoacrylates are also rigid (due to high reticulation density and the high strength of the adhesion leading to a rigid interface), not resorbable (slow degradation for long alkyl chains, but generation of formaldehyde and alkyl cyanoacetate upon degradation due to a reverse Knoevenagel in the presence of water), generate skin irritations (allergies), and have a high risk of carcinogenicity and embolism [220,225]. So, even if it is approved by the FDA for wound closure, it is worth noticing that formaldehyde and alkyl cyanoacetate are histotoxic (i.e., able to destroy the tissue). In the case of 2-octyl-cyanoacrylate, the peeling happens before the generation of the histotoxic products. On the other hand, surgeons claim its benefits, such as quicker procedures, lower inflammatory reactions, and better esthetic consequences (less scarring) as compared to staples and stitches [59]. The price of such adhesive is $50–100/5 mL. This is 100 times the price of a regular suture [191]. In addition, because of their high reactivity toward water, they have to be stored in air/water packages. On the other hand, they do not need preparation steps, they are ready to be used.

**Figure 22:**
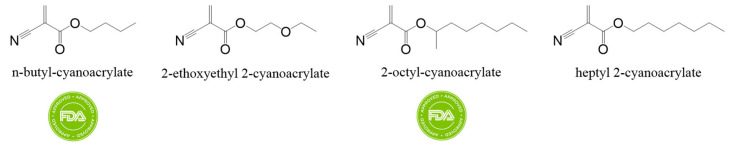
Formula of the different cyanoacrylate molecules used in surgical glues.

Additionally, due to its efficient use as wound closure adhesives, cyanoacrylates (CA) started to be used for internal surgery. The structure of cyanoacrylate glue is based on an acrylate with a long alkyl chain and a cyano group. The molecules used for dentistry, surgery, and veterinary medicine are butyl-CA, heptyl-CA, octyl-CA, and ethoxy ethyl-CA. Mainly because they are more hydrophilic, less toxic, have better mechanical properties, and generate less heat than the other CA. Indeed, CA with a short side chain and with low molecular weight allows for fast curing and quick degradation of the resulting polymer. The US FDA has only approved butyl and octyl CA, which are used in the US primarily for external use. In other countries, these adhesives have also been approved for internal use. As they have sealant and hemostatic properties as well as antimicrobial activity [22], they are used in some general surgical procedures such as periodontal, ophthalmic, facial, abdominal and pulmonary surgery (in addition to fibrin), as well as in laparoscopy. They are also utilized in vascular or lung surgery as sealant. (2015) It is also possible to use it for endoscopic injection in order to close fistulae or varices (in conjunction with oil). (2013) They are used to bond skin, bones, or teeth to tissues. For instance, Omnex™—Ethicon, a 2-octyl-cyanoacrylate, was the first FDA-approved cyanoacrylate for internal use in 2010 (Figure 22) [226]. This glue is used for vascular surgery. GEM (Glubran2) and Peters surgical (Ifabond) synthesized n-butyl-cyanoacrylate and n-hexyl-cyanoacrylate. Glubran2 is allowed in the European Union for surgery (laparoscopic incisions and digestive tract endoscopy). To sum up, these adhesives are as polyvalent for medical uses as for everyday life uses. They allow fast curing and a strong adhesion (except on wet tissues). Their main drawbacks are the exothermicity and the slow degradation rate, with toxic degradation products.

Not diluted, these glues remain cytotoxic. In addition, the exothermic reaction and the release of toxic monomers upon polymerization limit their use in a wide range of applications. That is why their use is mostly limited to topical applications.

##### C. Polyethylene Glycol Hydrogels

Poly(ethylene glycol) (PEG) is a polymer widely used in medical devices due to its water solubility, biocompatibility, and minimal immunogenicity. There are three main types of PEG-based surgical adhesives: photopolymerizable systems (e.g., ultraviolet-curable) and chemically crosslinked hydrogels (e.g., PEG/trilysine and functionalized PEG mixtures) [227].

**Figure 23:**
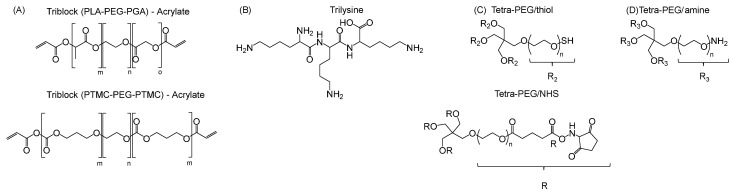
Formula of the component of the different PEG-based surgical glue (**A**): Focalseal^®^, (**B**): Duraseal^®^, (**C**): Coseal^®^, (**D**): SprayGel)^6^ (m, n, o, p: nb of repeating units).

Photopolymerizable PEG adhesives were first reported in 1993 and are based on PEG–PLGA (poly(lactic-co-glycolic acid)) copolymers synthesized via ring-opening polymerization of lactic and glycolic acids initiated by PEG [228]. These polymers are then functionalized with acrylate groups to allow photopolymerization. In aqueous environments, these triblock copolymers self-assemble into micelles, concentrating acrylate groups in the hydrophobic domains and enabling rapid polymerization—a process similar to polymerization-induced self-assembly (PISA). This type of gel is used as a sealant in lung surgeries to prevent air leakage. The glue is priced at $200–500 for 1–5 mL, offers an adhesion strength of 0.1–1.0 MPa, and has a viscosity ranging from 0.5 to 5.0 Pa·s. PEG–PTMC (poly(trimethylene carbonate)) offers better mechanical properties and is also used as a surgical adhesive. PEG–PLGA and PEG–PTMC can be used in combination, as in the surgical glue FocalSeal^®^ (Focal Inc., Helsinki, Finland), which received CE marking in 1999 and FDA approval in 2000 (Figure 23A) [229]. It is used alongside conventional closure techniques to prevent leaks in visceral and pleural surgeries. The hydrogel is formed under visible light (470–520 nm) in the presence of a photoinitiator (e.g., eosin Y). However, the presence of free radicals can limit its use in certain anatomical regions. This surgical glue is physiologically degradable via hydrolysis of ester groups, yielding safe byproducts such as PLA, PGA, PEG, and PTMC, which are eliminated via the kidneys.

The second type of PEG-based sealant was approved by the FDA in 2005 and involves a combination with trilysine [230]. Commercialized as DuraSeal^®^ (Integra, $500–1000 for 5 mL), it is delivered in a dual-syringe system: one syringe contains trilysine, tetraamine, and a basic buffer solution, while the other contains a four-arm PEG (pentaerythritol PEG) end-capped with N-hydroxysuccinimide (NHS) glutarate (Figure 23B). This product is used as an adjunct to sutures to prevent cerebrospinal fluid leaks in dural surgery, a common complication in this type of procedure. A crosslinked network is formed through amide bond formation between the NHS-activated PEG and the amino groups of trilysine, tetraamine, and endogenous proteins. The degradation products, PEG and amino acids, are eliminated via renal clearance. In addition to neurosurgery, this adhesive is also applicable in ophthalmic and vascular procedures. Its adhesion strength ranges from 0.1–1.0 MPa, with a viscosity of 0.1–5 Pa·s.

The third type of PEG-based sealant is commercialized by Baxter under the name Coseal^®^ (Figure 23C). It consists of two PEG solutions, dilute hydrochloric acid, and a sodium phosphate/sodium carbonate buffer. One syringe contains PEG-NHS, while the other contains thiol-terminated four-arm PEG. A hydrogel forms via the generation of thioester bonds, and disulfide bridges allow for transamidation with biological amines. This formulation polymerizes and crosslinks within 5 s, bonding to tissue, synthetic grafts, and itself. Coseal is used in Europe in combination with hemostatic agents for vascular and cardiac surgeries, as well as to prevent air leaks in lung surgeries. It remains in place for 7 days and is completely resorbed within a month. It costs $500–1000 for 2 mL, with a viscosity of 0.1–1.0 Pa·s and an adhesion strength of 0.1–1.0 MPa. Both thioesters and glutarate esters undergo hydrolysis. A major drawback of amide- and thioester-based sealants is their swelling capacity, which can render them unsuitable for certain applications (e.g., to avoid nerve compression).

SprayGel^®^ (Covidien) is another PEG-based adhesive using tetra-PEG/NHS and tetra-PEG/amine instead of thiol (Figure 23D). The amine-crosslinked network offers greater mechanical strength than its thioester counterpart. This adhesive is used in gynecological and colorectal surgeries.

In summary, these PEG-based sealants are biocompatible, physiologically degradable, and capable of forming covalent bonds with tissues even in wet environments. They are also flexible. However, their main drawbacks include hydrolytic degradation, which requires prior reconstitution (typically from lyophilized form in buffer solutions), and their short working time, which limits the possibility of repositioning tissues. Despite being excellent sealants, they exhibit poor mechanical properties and thus cannot be used as standalone adhesives but only as adjuncts to sutures. Their high cost must also be considered [24].

##### D. Conclusion

Synthetic surgical glues such as polyurethane, cyanoacrylate and PEG-based represent a significant breakthrough. Indeed, they offer tailored solutions to various clinical needs. PU exhibits high flexibility and durability. On the other hand, although it is rapid and has strong adhesion, cyanoacrylate shows poor flexibility and major irritation potential. In any case they are the primary solution for emergency sutures. PEG, on their side, has biocompatibility and physiodegradability, making them suitable for short-term applications (Table 4).

## 6. Conclusions and Perspectives

Surgical adhesives have become an increasingly vital component of modern medicine, offering less invasive alternatives to traditional sutures and staples for wound closure, hemostasis, and implant fixation. This review has explored various adhesion mechanisms, including chemical cross-linking and physical interactions with tissue, as well as methods for evaluating adhesive performance. We discussed recent developments in adhesive technologies, ranging from pressure-sensitive adhesives and wound dressings to surgical glues, with a particular focus on implantable adhesives.

Implantable adhesives derived from proteins, polysaccharides, and biomimetic materials show broad surgical potential due to their biocompatibility and biodegradability. However, challenges remain—most notably the need for precise control over degradation kinetics. Rapid degradation can undermine tissue stability before healing is complete, while slow degradation may trigger chronic inflammation. Additionally, their adhesive strength often falls short compared to synthetic alternatives such as cyanoacrylates.

Nature-inspired adhesives, mimicking mechanisms seen in mussels or geckos, demonstrate promising wet adhesion and enhanced biocompatibility. Yet, clinical adoption remains limited by high production costs, scalability issues, and incomplete understanding of their interactions with biological tissues—particularly concerning scar integration and long-term immune response.

Conversely, synthetic systems such as cyanoacrylates, polyurethanes, PEG-based adhesives, and acrylate/acrylamide formulations continue to be widely used for specific procedures. These materials offer strong adhesion, tunable biodegradation, and adaptability to surgical needs. However, they are not without drawbacks: cyanoacrylates can induce local toxicity or inflammation near sensitive tissues, while polyurethanes and PEGs may generate reactive degradation byproducts.

The integration of natural biopolymers with advanced synthetic materials represents a promising strategy to improve safety and efficacy. Future advancements will likely focus on four key areas: tailored performance for different surgical applications, improved biodegradability, enhanced adhesive strength, and minimized postoperative inflammation. The convergence of biomimetic design and synthetic chemistry holds great potential to meet the complex demands of procedures such as vascular, orthopedic, and reconstructive surgeries.

While current surgical adhesives—both natural and synthetic—have demonstrated substantial utility in clinical practice, their limitations in terms of precision, responsiveness, and integration with complex biological environments underscore the need for next-generation materials. In recent years, research has shifted toward more dynamic, multifunctional systems that can not only adhere but also interact intelligently with surrounding tissues. These emerging technologies—driven by advances in synthetic biology, bioinspired chemistry, 3D printing, and stimuli-responsive systems—represent a paradigm shift in adhesive design. The following section explores these innovative approaches, which aim to overcome the shortcomings of traditional adhesives and offer more personalized, adaptable solutions for modern surgical challenges.

Beyond these strategies, several emerging areas of research in surgical adhesives are gaining momentum. Innovations based on synthetic biology, bio-inspired underwater adhesion, 3D printing, and responsive systems are opening up new possibilities for adapting adhesive performance to complex and dynamic surgical environments. Synthetic biology is a powerful platform for the next generation of surgical adhesives [1,2,3,4]. It enables the production of bio-inspired proteins with customizable adhesion, degradation, and bioactivity profiles. These systems overcome the limitations of animal-derived glues and offer new opportunities for intelligent, multifunctional, and scalable solutions in clinical practice. Continued advances in protein engineering and biosynthetic manufacturing are expected to improve the performance and accessibility of these adhesives even further. Recent developments include the creation of engineered bicomponent adhesives based on protein aldimine condensation, which demonstrate enhanced adhesion strength and biocompatibility [5]. Furthermore, these biosynthetic systems are being explored for their potential to minimize tissue damage and infection risk as well as for their use in sealing air or fluid leaks [6]. Although synthetic biology-based adhesives have demonstrated promise in various fields, including ophthalmology (where PEG derivatives exhibit low toxicity and high performance, as reported by Guhan et al.) [7], neurosurgery, orthopedics, and cardiovascular surgery, further clinical research is required to ensure their safe and effective implementation [8]. Bioinspired underwater adhesives that mimic mussel foot proteins (MFP) and sandcastle worm secretions, for instance, offer a promising strategy for achieving strong, durable adhesion in wet surgical environments while offering biocompatibility and tunable degradability rates [9,10,11,12,13]. These systems exploit catechol-based chemistry, ionic complexation, or microstructural interlocking to maintain adhesion in the presence of blood and body fluids [14]. While clinical translation remains limited due to high costs and catechol oxidizable behavior, advances in synthetic analogs and production methods are rapidly bridging the gap between laboratory research and clinical application. Recent research has demonstrated the potential of hybrid systems that combine natural and synthetic components, such as silk, polydopamine, and Fe^3+^ ions, to replicate mussel and barnacle adhesion strategies. These systems can achieve high performance bonding in both dry and wet conditions [15]. Some of these adhesives are designed with environmentally triggered setting mechanisms, such as pH or temperature shifts, to replicate the controlled solidification processes observed in marine organisms [16,17]. This improves their usability in surgical contexts further [18]. Beyond surgery, these advanced adhesives are also showing promise in other biomedical applications, such as wound dressings and bone adhesives, where strong wet adhesion and biocompatibility are essential [19]. 3D printing technologies offer unparalleled opportunities for the fabrication of surgical adhesives with patient-specific geometries and spatially controlled properties [20]. By formulating bioinks containing adhesive groups (catechol, NHS, and acrylate) and functional polymers (PEG, PU, gelatine, alginate, chitosan, etc.), researchers have developed printable hydrogels that can adhere to complex tissue surfaces and deliver localized therapeutic functionality. Although still in the early stages of development, 3D printed glues could facilitate customizable wound sealing, in situ bioprinting, and smart surgical interfaces. On the other hand, careful attention must be paid to the viscosity and rheological properties of these formulations to ensure optimal printability, adhesion, and integration with biological tissues. Recent developments in 3D printing technology have enabled the production of programmable bioadhesive patches with tunable architectures that can be tailored to specific clinical needs [21]. Printable nanocellulose-based hydrogels are also being explored for their hemostatic and wound-healing properties, particularly in biodegradable formats suitable for tissue engineering [22]. These advances overcome the limitations of conventional sutures and staples, and are being increasingly adopted in surgical fields such as craniofacial, oromaxillofacial, and cardiothoracic surgery to produce patient-specific implants [23]. As 3D printing technologies become more accessible and cost-effective, integrating them into the development of surgical glue could significantly improve patient outcomes and expand therapeutic possibilities.

Stimuli-responsive surgical adhesives, also known as intelligent glues, are a promising development in personalized medicine. These materials can dynamically adjust their adhesion, degradation, or drug release profiles in response to specific physiological or external triggers, such as temperature [4,24,25], pH levels [26,27,28], enzymes [29], or light [30,31,32]. By enabling controlled behavior at specific sites and times, these glues can enhance tissue compatibility, reduce inflammation, and support complex procedures such as minimally invasive surgeries or targeted wound healing. Recent studies have shown that such adhesives can support advanced functions such as on-demand detachment and smart drug delivery systems [33,34]. This makes them ideal for use in wound closure and in cardiovascular and gastrointestinal surgery or for achieving liver hemostasis [35]. Some formulations even incorporate antimicrobial or self-healing properties, which can further improve post-surgical outcomes [34]. While these innovations offer significant advantages over traditional fixation techniques, challenges remain in translating them into routine clinical use [35,36]. Together, these emerging technologies represent a paradigm shift in surgical adhesive design, moving away from passive materials toward smart, bioengineered systems that can respond to the physiological challenges of modern surgery. Continued development of these technologies promises to enhance surgical outcomes, reduce complications, and expand their clinical applications in the years to come. Together, the evolution from conventional adhesives to smart, bioengineered systems illustrates the transformative potential of interdisciplinary innovation in surgical care. By integrating biocompatible design with advanced functionalities such as responsiveness, antimicrobial activity, and personalized application, these next-generation adhesives promise to address the limitations of current materials and significantly improve patient outcomes across a wide range of surgical specialties. Continued research, clinical validation, and scalable manufacturing will be key to translating these promising technologies from the laboratory to routine clinical use.

## Figures and Tables

**Table 1:** Possible acrylate low T_g_ segments (blue) and high T_g_ segments (red).

Monomer	2-ethylhexyl acrylate	N-octyl acrylate	N-butyl acrylate	Ethyl Acrylate	Methyl Acrylate	Vinyl Acetate	Acrylic Acid	Acrylamide
Formula	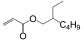	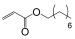	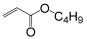	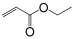	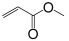	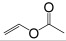	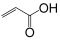	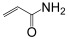
Tg of homopolymer (°C)	−70	−65	−54	−24	−6	30	106	179

**Table 2:** Features of fibrin-, gelatin-, and albumin-based surgical glues.

	Technology	Surgical Used Reported	Adhesion kPa	Price $/mL	Approval	Storage Condition (°C)	Instruction	Degradation in Physiological Condition (Body)	Commercial Name (Example)	Ref.
Sealant Hemostat Adhesive	Fibrine	1970	5–20	53–159 ^a,b^	FDA 1998	Freezer (−20)	Warm up at 37 °C	>1 month	Tisseel (Baxter)	[70]
Adhesive	GRF ^c^	1960	1–3	60–120	NO			2 months	Cardial (LeMaitre vascular)	[24]
Adhesive	GRFG ^d^	1967			NO		Warm up at 45 °C	>2 months	GRF (Microval)	[52]
Hemostat Adhesive	Gelatin + Thrombin	1970		62–100 ^a,b^	FDA 1999	RT (2–25)		4–6 weeks	Floseal (Baxter)	[28]
Adhesive	Gelatine + mTG	2004	12–23		CE 2011	Fridge (2–8)	Warm up at 37 °C	4–8 weeks	LifeSeal™ (Tiko-LifeBond)	[84]
Sealant Adhesive	Albumin + glutaraldehyde	1996	10–50	50–190 ^a,b^	FDA 2001	RT		Weeks to months	Bioglue^®^ (Cryolife)	[71]
Adhesive	Albumin + polyaldehydes	1780		300 ^a^	FDA 2017			6–8 weeks	PreveLeak (Baxter)	[23]
Adhesive	Albumin + PEG-NHS	1994	15–25	100–200	FDA 2010	Fridge (2–8)	-	<14 days	Progel™ (Davol)	[100]

^a^ https://medexsupply.com/, ^b^ https://surgishop.com/, ^c^ Gelatine/Resorcinol/Formaldehyde, ^d^ Gelatine/Resorcinol/Formaldehyde/Glutaraldehyde.

**Table 3:** Methods of bonding and their purposes for the species depicted in Figure 19.

Species	Way of Bonding	Purpose
bee	chemical (propolis)	nesting
caddishfly	chemical (silk)	nesting/hunting
chameleon	chemical (saliva)	hunting
dandelion	chemical (nectar)	mating (pollen spreading)
frog	chemical (mucus)	defence
ivy	chemical (polysaccharides glycoproteins)	attachement
mussel	chemical (catechol)	attachement
orchid	chemical (nectar)	mating (pollen spreading)
sea cucumber	chemical (mucus)	defence
silk worm	chemical (silk)	nesting
slug	chemical	defence/locomotion
spider	chemical (silk)	hunting/locomotion
velvet worm	chemical (mucus)	defence/hunting
gellyfish	chemical (nematocytes) physical (bar; cnidocytes)	defence/hunting
gecko	physical (spatulae)	defence/locomotion
nautilus	physical (suckers)	hunting/mating
octopus	physical (suckers)	attachement
see urchin	physical	attachement/locomotion
star fish	physical (suckers)	attachement
venus flytrap	physical (trap)	hunting

**Table 4:** Overview of biological, synthetic, and bioinspired tissue adhesives.

Adhesive/Family	Structure/Inspiration	Key Mechanism/Component	Main Function/Properties	Clinical Application	Regulatory Status	Pros	Cons
Fibrine glue	Plasma protein (fibrinogen)	Enzymatic polymerization (thrombin)	Rapid hemostasis Sealant Adhesive	-Wound closure -Skin graft -Various surgeries	FDA approved 1998	Biocompatible Biodegradable Support cell growth	High cost Low adhesion Risk of bloodborn pathogen transmition Application process
Gelatin–Resorcinol– Formaldehyde (±glutaraldehyde)	Collagen derivative	Aldehyde condensation	Adhesive	Various surgeries		Biocompatible biodegradable Support cell growth Strong cohesion Cost effective	Toxicity of the aldehydes
Gelatin-thrombine	Gelatin + enzyme	Enzymatic activation	Hemostasis Adhesive		FDA approved 1999		Moderate cohesion Foreign body reaction
Gelatin-mTG	Gelatin	Microbial transglutaminase	Enzymatic crosslinking adhesive	Gastrointestinal surgery	CE mark 2011	Good tissue compatibility	Low adhesion Poor mechanical strenght
Albumine–Aldehydes	Protein + aldehydes	Schiff base reaction (covalent bonding)	Sealant Adhesive	Vascular and cardiac surgeries	FDA approved 2001: glutaraldehyde 2017: polyaldehyde	Enhanced adhesion (vs. fibrine)	Potential cytotoxicity Low viscosity Inflammatory
Albumine–PEG	Protein + polymer	Chemical crosslinking	Prevent air leakage Adhesive	Lung surgery	FDA approved 2010	Low immunogenicity Degradable	Moderate adhesion High price Strorage condition
Chitosan	Polysaccharides	Hydrogen bonding Electrostatic interactions	Mucoadhesive Hemostasis	Wound healing Tissue adhesives		Biocompatible Physiodegradable Antibacterial	
Dextran based adhesives	Polysaccharides	Oxidation/aldehyde formation	Hemostasis		FDA: Conditional approval	Bioresorbable Enhanced adhesion (vs. fibrine)	Toxicity of aldehydes Foreign body reactions
Alginate	Polysaccharides	Ionic crosslinking	Sealant	Wound dressing Bones reconstruction Tissue regeneration	CE mark 2017	Low cost Biocompatible Enhanced adhesion (vs. fibrine)	Not biodegradable
Mussel inspired	DOPA rich proteins	Catechol Strong covalent/non-covalent bonds	Wet adhesion		Not commercialized	AdhesionWater insoluble Degradable	Extraction of natural protein not scalable and economical Toxicity suspected Storage of catechol
Gecko inspired	Microscopic spatulae	Van der Waals forces	Dry adhesion Hemostasis	Vascular surgery	CE mark 2017	Reversible/reusable Biocompatible Biodegradable	Low adhesivity wich deteriorates in wet conditions
Slug/snail mucus inspired	Viscoelastic secretion	Physical + chemical interactions	Tough Extensible wet adhesion			Resist to humidity	
Polyurethane based	Synthetic polymer	Chemical crosslinking	Adhesive	Abdominal surgery	FDA approved 2015	Strong cohesion Biocompatible Biodegradable	Toxicity of isocyanates Long curing time
Cyanoacrylate	Acrylate monomer	Anionic polymerization	Adhesive Selant Hemostasis	Wound closure Dentistry Surgery	FDA approved Wound dressing: 1998 Internal surgery: 2010	Very strong adhesion even in wet conditions Antimicrobial activity polymerize quickly	Brittle Tissue toxicity Not resorbable Exothermic
PEG-based hydrogel	Hydrophilic polymer	Chemical crosslinking	Sealant	Lung surgery Ophtalmic Vascular	FDA approved 2000	Customizable Bio-inert/bioresorbable matrix	High cost Poor mechanical properties
